# Quality by Design and Process Analytical Technology for On-Demand Drug Manufacturing Through 3D Printing

**DOI:** 10.3390/pharmaceutics18080935

**Published:** 2026-07-29

**Authors:** Imola-Rebeka Turac, Tibor Casian, Sonia Iurian, Alina Porfire, Rareș Iovanov, Daniela Elena Popa, Ioan Tomuță

**Affiliations:** 1 Department of Pharmaceutical Technology and Biopharmacy, Faculty of Pharmacy, University of Medicine and Pharmacy “Iuliu Hatieganu”, 41 Victor Babeș Street, 400012 Cluj-Napoca, Romania; imola.rebe.turac@elearn.umfcluj.ro (I.-R.T.); sonia.iurian@umfcluj.ro (S.I.); aporfire@umfcluj.ro (A.P.); riovanov@umfcluj.ro (R.I.); tomutaioan@umfcluj.ro (I.T.); 2 Department of Drug Control, Faculty of Pharmacy, “Carol Davila” University of Medicine and Pharmacy, 37 Dionisie Lupu Street, Sector 2, 020956 Bucharest, Romania; daniela_popa@umfcd.ro

**Keywords:** pharmaceutical 3D printing, personalised medicine, Quality by Design, Process Analytical Technology

## Abstract

Additive manufacturing, also known as 3D printing (3DP), is intended to enable personalised medicine by producing drug products on demand at the Point of Care (PoC), with dose, drug-release profile, and geometry tailored to the individual patient. Despite its promise, widespread adoption is limited by the absence of ready-to-use quality control (QC) methods for printlets at the PoC. Process Analytical Technology (PAT) tools, particularly vibrational spectroscopic methods like Near-Infrared and Raman spectroscopy, can offer real-time monitoring to ensure the safety and consistency of printed dosage forms. Integrating these tools within a Quality-by-Design (QbD) framework can enhance process understanding, control variability, and minimise risk. Regulatory implementation and technological innovation remain essential for the broader clinical implementation of 3DP in pharmaceutical manufacturing. This review presents an overview of currently existing studies on PAT tools explored for non-destructive quality control across 3DP techniques, examines the correlation between Critical Process Parameters (CPPs), Critical Material Attributes (CMAs), and the Critical Quality Attributes (CQAs) of 3D-printed dosage forms within a QbD context, and outlines the current regulatory landscape alongside key limitations and future directions for the broader integration of 3DP into pharmaceutical development and manufacturing. Current evidence shows that PAT application remains uneven across printing technologies and is predominantly directed at final product quality control, rather than the real-time process monitoring required for a fully closed-loop QbD framework. Existing spectroscopic models are largely restricted to single formulations, printers, and APIs, and the absence of standardised validation reporting and transferability assessments represents a key barrier to routine implementation.

## 1. Introduction

Three-dimensional printing (3DP) has emerged as a promising approach for the fabrication of personalized pharmaceutical dosage forms at the Point of Care (PoC), enabling a paradigm shift from the conventional "one-size-fits-all" manufacturing model towards the on-demand production of individually tailored dosage forms adapted to patient-specific needs [1,2,3,4]. Owing to its inherent advantages, including dose flexibility, design versatility, and the ability to produce complex geometries and achieve customised drug release profiles, 3DP is increasingly attracting interest for personalised medicine [5,6].

The main challenges limiting the broader implementation of 3DP at the PoC relate to the limited availability of reliable quality control protocols, which are raising concerns over the safety and consistency of printed medicines. Since 3DP is intended for small-scale, individualised production, conventional destructive quality control methods, which necessitate duplicate fabrication and generate additional waste, are inefficient and unsustainable [5]. Therefore, regulatory bodies such as the Medicines and Healthcare products Regulatory Agency (UK MHRA), the U.S. Food and Drug Administration (FDA), and the European Medicines Agency (EMA) have initiated efforts towards the development and integration of Process Analytical Technology (PAT) tools for real-time quality control (QC) during the printing process [7]. The introduction of the regulatory framework for decentralised medicine at the PoC by the UK MHRA and the EMA guidance on Quality and Good Manufacturing Practice for 3D-printed solid dosage forms mark a major step towards the implementation of 3DP at the PoC [8]. Furthermore, there is a lack of regulatory clarity concerning the design and manufacturing processes, which must be addressed to enable safe and effective PoC implementation [9].

According to the FDA, the primary aim of PAT is to enhance process understanding and control, while reducing uncertainty and variability in the quality of the final product [10]. This objective is closely aligned with the principles of the Quality-by-Design (QbD) framework, which plays a key role in achieving consistent and reliable product quality. To ensure the consistent quality of each individually manufactured unit, all sources of process variability must be well understood and managed. This begins with the definition of the Quality Target Product Profile (QTPP), which describes the prospective quality characteristics a dosage form should possess to ensure its intended safety and efficacy. The QTPP enables the selection of the Critical Quality Attributes (CQAs), the physical, chemical, or biological properties of the final product that must remain within defined limits to guarantee the required quality, and guides the identification of Critical Process Parameters (CPPs) and Critical Material Attributes (CMAs) that must be systematically controlled [2,11]. In essence, the QTPP defines the quality goal, the CQAs specify the measurable product properties that define that goal, and the CPPs and CMAs represent the process and material factors, respectively, that must be controlled to achieve it.

These PAT tools are useful to monitor the pre-established CQAs and known CPPs to ensure complete control over the 3DP manufacturing process. Depending on the specific technology and requirements, PAT tools may be implemented at-line, on-line, or in-line to generate multivariate datasets that can be exploited using chemometric and Multivariate Data Analysis approaches [7]. The most widely used PAT tools are based on vibrational spectroscopic techniques, such as Near-Infrared Spectroscopy (NIRS) and Raman spectroscopy. These non-destructive analytical methods provide real-time physico-chemical information about the dosage units, require minimal human intervention, and involve minimal to no sample preparation [5,12,13,14,15]. Thus, they are ideal for the QC of 3D-printed pharmaceuticals, where each dosage unit can be unique in terms of design and drug load.

Despite the growing body of literature on pharmaceutical 3DP, the majority of published studies continue to rely on destructive end-product analysis, and the incorporation of PAT tools remains the exception rather than standard practice. Existing reviews have characterised the CPPs and CMAs governing printlet quality in considerable detail and established their relationships with the corresponding CQAs [7,16,17,18,19]. However, these parameters have been examined primarily from a process-development perspective, and the extent to which each is actually addressed by a dedicated PAT method has not been systematically assessed. Consequently, it remains unclear which quality-determining parameters are currently supported by validated non-destructive monitoring and which fall outside existing analytical coverage—a distinction with direct implications for real-time release and routine QC at the PoC. This article reviews the principal achievements and current developments of PAT tools in the pharmaceutical domain, with an emphasis on their integration into 3DP workflows. It further explores the process parameters that significantly influence the CQAs of 3D-printed dosage forms, aiming to highlight why the advancement of non-destructive, real-time quality control technologies is critical to ensure the reliability and reproducibility of personalised medicines produced via 3DP. In addition, it outlines the most relevant PAT tools applicable to QC in pharmaceutical 3DP and synthesises the existing literature on PAT implementation within 3DP QC. It also outlines the current regulatory landscape, highlights key limitations, and discusses prospects of pharmaceutical 3DP.

## 2. Quality-by-Design Elements in Pharmaceutical 3D Printing

Currently, there are seven 3DP technologies in pharmaceutics, which can be organised into four major groups, based on their mechanism: nozzle-based deposition systems (Fused Deposition Modelling, Semisolid Extrusion, and Direct Powder Extrusion), jet-based systems (Inkjet Printing and Binder Jetting), laser-based systems (Selective Laser Sintering), and light-based systems (Stereolithography) [9]. Table 1 presents the 3DP technologies used in pharmaceutics and summarises the main advantages and disadvantages of each technique. It is important to note that this article does not aim to extensively review pharmaceutical 3DP techniques. This review identifies the most relevant CPPs and CMAs for each 3DP technology and relates them to the CQAs they impact, with particular attention to which of these parameters are currently addressed by PAT tools and which remain without analytical coverage—a critical gap in the implementation of QbD-driven development of 3D-printed dosage forms. For a detailed description and comparative analysis of the 3DP techniques, readers are referred to existing comprehensive reviews in the current literature [20,21,22].

The crucial CQAs of 3D-printed dosage forms are mass uniformity, drug content uniformity, dissolution profile, mechanical properties, and dimensional accuracy. Additionally, printing resolution, porosity, and solid-state of the API can also be important CQAs [6,23]. In the following, a brief description highlights each printing method and discusses the crucial CPPs and CMAs that affect the main CQAs of the 3D-printed dosage forms.

**Table 1:** Main advantages and limitations of pharmaceutical 3DP technologies.

3DP Technology		Advantages	Inconvenients	References
Nozzle-based printing technologies	FDM	Small, less expensive equipment Allows the creation of complex geometries No organic solvent required Any formulation design can be achieved A great variety of polymers that allow tailored drug release Through HME, the solubility and bioavailability of poorly soluble drugs can be increased Good patient acceptability Allows the formulation of polypills	High heating temperatures can lead to API degradation Limited use of thermolabile APIs Fewer polymers available for immediate release Limited drug loading can be achieved Printlet quality depends on filament quality Two-step process	[9,18,24,25,26,27,28,29,30,31]
	DPE	Avoids formulation and preparation of filaments	Produces low-mechanical-strength dosage forms Less amount of adequate polymers available High extrusion temperatures needed	[32,33,34,35,36]
	SSE	Allows high API loading in printed dosage forms Offers versatility in excipient selection Low printing temperatures → suitable for thermolabile APIs Enables portability Easy elaboration of ready-to-use printing cartridges No cross-contamination due to the use of disposable syringes Few processing steps	Produces dosage forms with low mechanical strength Need for post-printing drying Complete solvent evaporation is needed Risk of shape loss	[28,32,37,38,39,40,41,42]
Laser-based printing technologies	SLS	High-resolution printing method Lack of solvent use Few processing steps	Possible material degradation due to the laser Excess powder is needed for printing No powder mixing during the process Could induce fluorescence Offers lower dimensional accuracy	[18,43,44,45]
Light-based printing technologies	SLA	High-resolution printing technique No need for high temperature Ability to print micro-sized structures	Reduced number of pharmaceutical-grade suitable excipients Requires a photopolymerisable liquid resin as printing ink Lack of resins that give soluble matrices Incompatible with UV-sensitive drugs Inadequate printing speed	[16,18,21,43,46,47]
Jet-based printing technologies	IJP	Can achieve high printing speed High-resolution printing technique Does not require high molecular weight polymers Possibility to print on porous/non-porous/biodegradable films Suitability for short-term production Formation of API polymorphs due to applied heat (TIJ printing) High printing precision → high droplet precision in DoD; large substrate variability; highly versatile in formulation design	Susceptibility to ink bleeding Binder oversaturation UV irradiation → API degradation Limited ink variability (solutions/nano-suspensions) Lack of suitable solvent evaporation technique	[3,21,33,39,48,49,50]
	BJ	Extensively used in rapid prototyping High drug loading is possible (in the ink/solid)	Inefficient powder usage Need for post-processing steps Produces friable and brittle dosage forms Stability problems related to the use of organic solvents	[21,51,52]

API = Active Pharmaceutical Ingredient; BJ = Binder Jetting; DoD = Drop-on-Demand; DPE = Direct Powder Extrusion; FDM = Fused Deposition Modelling; HME = Hot Melt Extrusion; IJP = Inkjet Printing; SLA = Stereolithography; SLS = Selective Laser Sintering; SSE = Semisolid Extrusion; TIJ = Thermal Inkjet; UV = Ultraviolet.

### 2.1. Extrusion-Based Technologies

#### 2.1.1. Fused Deposition Modelling (FDM)

Fused Deposition Modelling (FDM) is an extrusion-based 3DP technique that produces tailored dosage forms by heating, melting, and extruding an API-loaded thermoplastic filament in a controlled layer-by-layer manner. FDM enables the fabrication of varied dosage-form geometries, with drug-release profiles tunable through controlled adjustments of the printing parameters. Its process flexibility and suitability for low-throughput manufacturing make it a strong candidate for small-scale pharmaceutical production [9,11]. Due to its flexibility, not only 3D-printed tablets, but also fast-dissolving oral films, suppositories, and implants have recently been gaining a lot of attention [33,53].

FDM requires the use of API-loaded filaments, which are most commonly produced by Hot Melt Extrusion (HME). In this process, a defined amount of API is blended with a polymer matrix, melted, and processed through a single- or twin-screw extruder to obtain suitable filaments for FDM printing [53]. Therefore, the CQAs of the printed dosage forms can only be achieved if the quality of the API-loaded filament is ensured. This depends on the control of key CMAs and CPPs during HME. Table 2 summarises the main CPPs and the CQAs they influence, along with the methods used for their monitoring.

The primary objective of HME in pharmaceutical FDM is to produce filaments with homogeneous API distribution, consistent diameter, and suitable mechanical and thermal properties—CMAs that directly determine printability and final product performance. Achieving these attributes requires selecting thermoplastic polymer carriers whose processing temperatures fall below the API’s degradation threshold. However, many pharmaceutical polymers possess glass transition temperature (Tg) or melting temperature values that exceed the thermal stability window of the API, making the incorporation of a plasticizer necessary to lower the polymer’s Tg and reduce melt viscosity, thereby enabling extrusion without compromising API integrity. A balance must be maintained between lowering the processing temperature and the amount of plasticizer added, as excessive plasticization can impair printability [6,54,55]. However, in some formulations, plasticizers such as mannitol may fail to fully melt during the printing process, leading to interlayer gaps and poor fusion, a phenomenon demonstrated by Jørgensen et al., where SEM images of tamoxifen-loaded printlets containing mannitol revealed distinct voids between deposited layers [5].

One of the main challenges in HME is to obtain a filament with a constant diameter (typically 1.75 ± 0.5 mm). Several CMAs and CPPs influence filament diameter. The most important HME process parameters, such as temperature, die swell, screw speed, feeding rate, and conveyor belt speed, are formulation-dependent, and process optimisation requires a considerable amount of time and materials [5]. An inconsistent filament diameter leads to either poor gear grip in the 3D printer or printer clogging [5,9]. Ponsar et al. optimised the extruder setup by incorporating a laser-based diameter measuring module, a conveyor belt, and a winder to achieve the desired and constant filament diameter. The diameter fluctuations of filaments were directly correlated with the powder flowability [56]. Filament diameter variations directly affect the mass uniformity of the printlets, as thicker parts of the filaments could extrude and deposit more melted material per time unit, compared to thinner filaments [56].

Material feeding rate and conveyor belt speed significantly influence the filament diameter. Specifically, an increased feeding rate tends to produce filaments with larger diameters, whereas a higher conveyor belt speed results in thinner filaments [56].

Screw speed also impacts filament characteristics: higher screw speeds are associated with reduced internal pressure within the extruder but lead to greater consistency in filament diameter. Additionally, extrusion temperature plays a crucial role in the quality of the filament. When the temperature exceeds the Tg of the polymer, the decrease in melt viscosity can lead to excessively thin filaments. Conversely, insufficient temperature that does not adequately melt the mixture may yield thicker filaments with inadequate mechanical properties [32,56]. Process temperature can influence both the stability and the solid-state of the API within the formulation blend. Therefore, the extrusion temperature should be carefully selected to preserve the optimal solid-state of the API, and it is typically maintained 20–30 °C below the degradation temperature of the API and excipients [57].

In FDM 3DP printing, temperature is an important parameter, influencing the solid-state of the API within the printed dosage forms, weight, porosity and drug content uniformity, as well as the drug release rate from printlets. As FDM relies on filament melting, it is crucial to work at high temperatures, which allow the mixture to melt and to be extruded. The high operating temperatures during FDM can positively influence the printlet quality by enhancing the polymer–drug interaction for the solid dispersion formation, ensuring an amorphized API, increasing the bioavailability and dissolution rates [17]. Enhancing the polymer–API interaction could lead to better bioavailability of the API. However, the high operating temperatures could change the physico-chemical stability of the API, resulting in degradation products, printlet shrinkage, or recrystallization, which could highly impact the quality of printlets, such as their drug release profiles and drug content [6,14,57]. Temperature can be reduced by using low-melting-point, immediate-release polymers, such as Eudragit^®^ E PO, PVP (polyvinylpyrrolidone), Kollidon^®^12 PF, or VA64, which melt below 100 °C, or the addition of plasticizers in the formulation. This way, FDM could also be used in case of thermolabile APIs [26].

Printing speed is another important process parameter influencing the mass uniformity and the dimensional accuracy of printlets. A higher printing speed and lower layer height improve the dimensional accuracy [58,59]. Alhijjaj et al. demonstrated that printing speed leads to printlet mass variation. A higher printing speed correlated with a higher mass variation [32]. Although a higher printing speed can reduce the printlet fabrication time, weight variation is considered to be of higher importance than the printing time, ensuring qualitative final dosage forms [58].

Infill percentage is a key CPP, affecting the mechanical properties, drug release profile of printlets, and the mass uniformity. Higher infill is correlated with a higher mechanical resistance, because of the higher printlet density [23,60]. Henry et al. studied the influence of the infill percentage on 3D-printed caplets loaded with zolpidem hemitartrate and concluded that mass uniformity, dissolution rate, and mechanical resistance were mainly influenced by the infill percentage. A lower infill percentage resulted in a weight decrease, lower mechanical resistance, and a higher dissolution rate [23]. Infill percentage also affects drug dissolution rate. The higher the infill density, the slower the dissolution rate is, as proved by Fanous et al. [61].

Nozzle diameter may also affect the quality of the printed product, as it determines the extrusion rate of the melted filaments. It can impact the mass of the printlet, but also its resolution and mechanical properties [62].

Shell/layer thickness and height are believed to affect the mechanical properties, drug release rate, and mass uniformity of the printlets [23,60,63]. Pietrzak et al. found that layer thickness affects printing resolution, as a lower printed layer thickness increased printing resolution, ensuring printlet mass uniformity [63]. However, Zhang et al. contradicted the previous statement, affirming that shell thickness had an insignificant influence on the mass uniformity of the printlets [60]. Layer height is believed to affect printlet hardness, since a thicker deposited layer needs more cooling time, increasing the time for interlayer bond formation, enhancing printlet hardness. Moreover, layer height affects printlet weight uniformity, since a higher layer height can increase printlet weight [23].

A less studied CPP, overlap showed to have an impact upon the final product quality, as it increased the bonding between the shells and the infill layers, contributing to an increase in layer adhesion, affecting the mechanical properties of the printlets. A better overlap leads to higher mechanical properties, due to better bonding between the layers [60].

#### 2.1.2. Semisolid Extrusion (SSE)

Semisolid Extrusion (SSE) is a 3DP technique that produces printlets by extruding a semi-solid mass (such as a gel or paste) from a syringe, using pressure. Mechanical pressure or pressurised air is gradually applied to the syringe until the semisolid material begins to flow through the nozzle. Pressure continues to increase until the material reaches a constant flow rate, to ensure drug load and mass uniformity of the printed dosage forms. Given that it is an extrusion-based technique, most CPPs are common with FDM process parameters, with the key difference lying in the CMAs, such as the rheological properties of the printing material, as shown in Table 2 [37].

In contrast to FDM, SSE 3DP is suitable for heat-sensitive drugs, biopolymers, and living cells, while enabling the fabrication of various dosage forms, including oral solid or chewable formulations, as well as rectal formulations [64]. The use of disposable pre-filled cartridges also makes SSE one of the first 3D printing techniques compatible with GMP requirements and highly feasible for Point-of-Care and academic pharmaceutical formulation [65,66,67,68,69,70].

A key challenge in achieving the desired dosage form CQAs is controlling paste properties, particularly the rheological behaviour, which strongly influences printlet quality.

Rheological properties of the paste (viscosity and swellable properties) affect the extrudability, layer adhesion, and thus affect the mass uniformity, the mechanical properties, and the dissolution profile of the printlets. The rheological properties of the paste are highly dependent on the characteristics of the excipients [41]. For instance, the incorporation of water-soluble excipients into aqueous-based pastes can produce low-viscosity systems that are unable to retain their shape after extrusion, thereby compromising mass uniformity and dimensional accuracy. Conversely, highly viscous pastes exhibit poor flowability and require high extrusion pressures, often resulting in filament deformation and nozzle clogging. Therefore, paste viscosity plays a critical role in extrudability and determines the extrusion pressure, directly affecting mass uniformity and dimensional accuracy of the printed products [39,40].

Studies have shown that gels exhibiting rapid and reversible viscoelastic behaviour are among the most suitable excipients for SSE, as they can be extruded at low pressure while retaining sufficient mechanical strength to preserve printlet shape and layer integrity [39].

Yield stress influences paste compressibility. It increases with particle aggregation and higher solid volume fractions, particularly when weak or insufficient attractive forces exist between the API and excipients [39]. Particle aggregation could be reduced by increasing the viscosity of the paste and improving polymer-particle attraction [39,71]. Yield stress affects the mechanical properties and dimensional accuracy of the printlets, as the semi-solidification process of the printlets is modelled by a yield stress buildup. A high or inconsistent yield stress may cause an incomplete layer formation, affecting the dimensional accuracy and mass uniformity. A low yield stress forms weak structures, affecting the mechanical properties of the printlets [39].

Extrusion pressure is one of the most important CPPs, affecting mass-, content uniformity, and dimensional accuracy. A sudden decrease in pressure indicates the presence of air inside the syringe, and a higher pressure indicates nozzle clogging [37,41].

Nozzle diameter is a CPP that affects extrusion pressure, mass uniformity, dimensional accuracy, and printing resolution. Smaller nozzle diameters provide higher printing resolution, resulting in greater printing precision and smoother surface morphology. In contrast, wider nozzles reduce printing pressure [39], improve filament consistency, and increase structural rigidity, thereby reducing collapse, mass variation, and printer-head clogging [41,72].

Printing speed is directly correlated with the extrusion pressure, as a higher printing speed increases extrusion pressure. Therefore, it impacts the flow rate of the semi-solid mixture, influencing the mass and content uniformity, as well as printing resolution. A higher printing speed leads to a decrease in the extruded filament diameter, due to the increase in shear- and volumetric flow rate, causing mass variations [39].

Extrusion temperature influences the solid state of the API, mass and content uniformity, mechanical strength, and dissolution profile [62]. Excessively high extrusion temperatures can over-fluidize the paste, leading to unintended leakage and uncontrolled deposition, compromising printlet quality. A properly selected extrusion temperature should ensure API amorphization and, consequently, adequate dissolution, while maintaining uniform mass and drug content, acceptable resolution, and suitable mechanical properties [41].

The extrusion head positioning in SSE 3DP is crucial for the dimensional accuracy and mass uniformity of the printed product. If the distance between the build plate and the nozzle is too high, extruded material may accumulate at the tip of the nozzle. In contrast, if the nozzle is too close to the build plate, the material flow could be inconsistent, leading to improper deposition, causing mass and design variations [41].

Other CPPs, common to SSE, include infill density and layer height, which are generally studied in extrusion-based printing technologies and were described above.

#### 2.1.3. Direct Powder Extrusion (DPE)

Direct Powder Extrusion (DPE) is a 3DP technique in which the powder blend is directly loaded into a heated nozzle, where it melts and is extruded layer by layer onto the build platform [33,62].

Since DPE is a single-step method, the properties of the powder mixture, such as particle size distribution, flowability, and packing density, are critical in obtaining good-quality printlets. The particle size of the powder affects the mechanical properties and the printing resolution of the printlets. The lower the particle size, the higher the packing density can be; therefore, the printlets will have higher mechanical strength and a better printing resolution [73]. Powder flowability greatly affects printing resolution, mass- and content uniformity. Since this printing technology relies on a layer-by-layer deposition of the powder, poor flowability reduces printing resolution, as particles tend to agglomerate. Also, an agglomerated powder deposition is most likely inhomogeneous, leading to content variations [74,75].

Moisture content and melt viscosity play a critical role in DPE, influencing the dimensional accuracy of printlets. Excessively high mixture viscosity and a highly moist blend may hinder the fabrication of the predefined 3D printlet design. Therefore, the best excipients for DPE are low-viscosity polymers, with lubricants and plasticizers, stored in airtight containers, which are preferred to ensure continuous deposition of the molten mixture and to achieve mass and content uniformity [62,76].

The most important CPPs, highlighted in Table 2, are printing temperature and printing speed [74]. Printing temperature is determined by the melt viscosity of the excipients used. As in FDM, thermally stable ingredients need to be used. Printing temperature affects extrudability, so the mass uniformity, printing resolution, and the dissolution profile as well [44,76]. Normally, the powder mixture should be heated 30 °C above the Tg of the mixture, so that the API would be amorphized, ensuring a good dissolution profile. Although in some cases a higher temperature can help the extrudability and printing resolution, it can also degrade the API or excipients and lead to content variations [62,76].

Printing speed affects the mean residence time of the mixture in the printer and the thickness of the extruded strand. It also influences the printing resolution and dimensional accuracy of the printlets. Excessively high printing speeds may cause smearing of previously deposited layers, resulting in shape and diameter variations and increased surface roughness [36,73].

Since it is a 3DP method based on extrusion, nozzle diameter plays an important role in the printing resolution, similarly to FDM and SSE.

**Table 2:** Main CQAs and CPPs/CMAs in FDM, SSE and DPE and their assessment through traditional vs PAT methods.

		CQA	CMAs */CPPs	Conventional Methods	PAT Testing Methods	References
**FDM**	**Intermediate product ** **(filament)**	Content uniformity	* Powder rheology Screw speed Screw configuration	HPLC	In-line UV–Vis spectroscopy [17,77,78] NIRS reflectance [79] Raman spectroscopy [80]	[17,72]
		Diameter	Conveyor belt speed Material feeding rate Screw speed Extrusion temperature * Die swell	Diameter measurement (digital calliper) Visual assessment	In-line size determination [55]	
		Mechanical resistance	Powder homogeneity Extrusion temperature	Texture analyzer	Acoustic emission technology [81]	
	**End product (printlet)**	Mass uniformity	Printing speed Filament diameter Shell/layer thickness Infill	Gravimetric measurement	Filament advancement detection sensor [82]	[25,28,32,72,83,84,85]
		Content uniformity	Filament diameter Printing temperature	UV spectroscopy/HPLC	NIRS reflectance [86] NIRS transmittance NIRS-HIS [5] Raman spectroscopy [80] Raman chemical imaging [87]	
		Dissolution profile	Infill Shell/layer thickness Printing temperature	UV spectroscopy/HPLC	NIRS/Raman mapping [59]	
		Dimensional accuracy	Printing speed Overlap	Diameter measurement (digital caliper)	-	
		Mechanical resistance	Infill density	Texture analyzer Crushing strength tester	Acoustic emission technology [81]	
		Printing resolution	Printing speed	SEM	-	
		Solid state properties of API	Printing temperature	XRPD DSC TGA	NIRS [88]	
		Porosity	Printing temperature	SEM	Terahertz Pulsed Imaging µ-CT [85]	
**SSE**		Mass uniformity	* Rheological properties of paste Extrusion pressure Printing speed Extrusion temperature Printing orientation	Gravimetric measurement	In-line pressure sensor monitoring [40] In-line analytical balance [89,90]	[37,41,66,67,91,92,93]
		Content uniformity	Extrusion pressure Printing speed	UV–Vis spectrophotometry HPLC	NIRS [94]	
		Dissolution profile	Yield stress Infill density Layer height	In vitro dissolution tester Disintegration tester	In-line pressure sensor monitoring [40]	
		Dimensional accuracy	Rheological properties of paste Extrusion pressure Printing speed Yield stress Extrusion temperature Printing orientation Infill density Layer height	Digital microscope Amplitude sweep tester Diameter measurement (digital caliper)	In-line pressure sensor monitoring [40]	
		Mechanical properties	Infill density Layer height	Crushing strength tester Friability tester	-	
		Printing resolution	Layer height Printing speed	SEM Low-vacuum SEM	-	
		Solid state	Printing temperature	XRPD DSC FTIR TGA	-	
**DPE**		Mass uniformity	* Powder rheological properties Printing temperature	Gravimetric measurement	In-line analytical balance [95]	[34,95]
		Content uniformity	Printing speed	HPLC	NIRS [38]	
		Dissolution profile	Printing temperature	In vitro dissolution tester	-	
		Dimensional accuracy	Printing speed	Diameter measurement (digital caliper)	-	
		Mechanical properties	* Powder rheological properties	Texture analyzer	-	
		Printing resolution	* Powder rheological properties Printing speed	SEM	-	
		Solid state	Printing temperature	DSC TGA XRPD FTIR spectroscopy	-	

* = CMAs; DSC = Differential Scanning Calorimetry; FTIR = Fourier-Transform Infrared; SEM = Scanning Electron Microscopy; TGA = Thermogravimetry; XRPD = X-Ray Powder Diffraction; µ-CT = Micro-Computed Tomography.

### 2.2. Jet-Based Technologies

#### 2.2.1. Inkjet Printing (IJP)

Inkjet Printing (IJP) is based on the precise deposition of API-containing inks onto a carrier substrate, enabling the fabrication of personalised dosage forms [14,16,50].

Based on the dispensing mechanism of the ink, the transducer used, the Drop-on-Demand (DoD) technique classifies into piezoelectric inkjet (PIJ) printing, thermal inkjet (TIJ) printing, and electromagnetic IJP [2,3,96,97]. PIJ printing relies on the deposition of ink droplets obtained via a mechanical deformation applied by a piezoelectric transducer on the ink reservoir, producing droplets by the vibration of a piezoelectric crystal in the firing chamber. TIJ printing uses a resistor that helps to rapidly form and expand a vapour bubble in the dispensing chamber, which forces a droplet of fluid out [7,14,98]. DoD can be classified regarding the substrate onto which the ink is deposited, as drop-on-drop or drop-on-solid technique [14,97].

The ink formulations for DoD Inkjet Printing include the API(s), solvents, cosolvents, humectants, and occasionally they can contain surface active agents, viscosity modifiers, and colourants. The selected excipients are based on the API’s physico-chemical properties, as the highest solubility needs to be attained [2,48].

Substrate type is a critical choice in IJP [48]. Substrates control drug release rate due to their physico-chemical variabilities, such as porosity, surface area, wettability, and hydrophilicity. Drug release rate also depends on the deposited ink–substrate surface interaction. The physico-chemical interaction between the substrate and the pharma ink may affect dimensional accuracy, mechanical properties and the resolution of the printed products [16]. A higher porosity and surface area contribute to a faster drug release, as a larger diffusion area is available (as shown in Table 3). Drug release rate is also faster if the substrate is hydrophilic, as well as the API, as it enhances the availability. Conversely, hydrophobic drugs deposited on hydrophobic substrates significantly reduce the dissolution rate. Wettability and porosity of the substrate can influence the adhesion rate of the pharma-ink, affecting the mechanical properties of the printed dosage forms [50].

Ink rheology plays a key role in IJP, influencing mass and drug content uniformity (as shown in Table 3). Key rheological parameters include viscosity, surface tension and inertia, which together determine the ink’s droplet formation and deposition behaviour. For optimal performance, the viscosity of pharmaceutical inks should typically range between 2 and 20 mPas. Excessively low viscosity compromises dimensional accuracy by uneven droplet spreading. On the other hand, excessively high viscosity can hinder proper droplet ejection and interlayer adhesion, ultimately compromising the mechanical integrity of the printed structures [99]. Thus, ink rheology and ink–substrate interaction are key CMAs in IJP that need to be further studied.

Jetting velocity is an important CPP (as shown in Table 3), which depends on the printing speed. Similarly to other printing techniques, a faster printing speed causes inadequate drying of the deposited layer, resulting in lower dimensional accuracy. Printing speed also affects the mechanical properties of the printed products. Higher printing speeds may reduce printlet mechanical strength due to increased inertial forces [16].

Nozzle diameter determines droplet size, affecting printing resolution. A wider nozzle typically produces larger droplets, while a narrower one allows for smaller, more precise droplets. The nozzle surface also matters. A hydrophobic inner surface tends to delay droplet release, which can reduce accuracy, while a hydrophilic surface can help ensure quicker and cleaner droplet formation [99].

Droplet spacing is influenced by how fast droplets are formed and ejected and affects the mechanical properties and dimensional accuracy. If the formulation is too viscous or the droplet detachment is slow, the droplets will be spaced further apart. This can affect how well the printed lines or structures come together. Proper synchronisation between droplet formation and the movement of the printhead is essential for consistent spacing and layer definition [50,99].

Droplet properties such as shape, velocity, and trajectory can affect the dimensional accuracy of printlets. Droplets with higher surface tension tend to move faster and are more stable, which is generally desirable. However, if the formulation is too viscous, droplet motion can slow down, and the shape may become less uniform [50,100].

Droplet volume depends largely on the nozzle diameter and printer actuation. The force and duration of each actuation pulse must be selected to match the formulation’s properties. Too little force may lead to incomplete droplet formation; on the other hand, too much can produce splashing or multiple droplets. Achieving consistent droplet volume is essential for uniform dosing and dimensional accuracy [50,100].

Jetting temperature plays a key role by modifying viscosity, as higher temperatures improve flow and droplet formation but may degrade sensitive compounds, while low temperatures can hinder jetting. Hence, viscosity variation may lead to mass variations, but also dimensional variations.

#### 2.2.2. Binder Jetting (BJ)

Binder Jetting (BJ) is a drop-on-solid or drop-on-powder 3DP technique, where layers are created from powder bed solidification due to a binding solution extruded from the printer nozzle [64,74,101].

The efficiency of the BJ 3DP process, as well as the attainment of CQAs (as shown in Table 3), largely depend on the jetting performance of the binder solution and the properties of the powder mixture. The most important characteristics of a powder blend intended for Binder Jetting include good flowability, sufficient packing density, and a uniform particle size distribution, all of which are essential for intact and consistent layer forming [44,102].

The CMAs of the binder solution involve appropriate viscosity, density, and surface tension. The binder must ensure complete wettability of each powder layer deposited and ensure adequate binding. The binding agent must not chemically interact with the properties of the API [73].

Binder saturation and layer thickness affect the mechanical properties of the printlet, as a higher layer thickness could be harder to wet by the binder liquid [73]. According to Vaezi et al., layer thickness considerably increased the tensile strength in the printed products [103].

Another CPP is the roller speed and the spreader speed, as these influence the mass and content accuracy of the printlets. A higher roller speed increased printing accuracy [73,102].

Printing speed impacts dimensional accuracy and printing resolution. A higher printing speed leads to inaccurate solvent evaporation from the previous layers and increases surface roughness, eventually causing pre-printed layer smearing [73,104].

The orientation of the printed object influences the mechanical properties of the printlet. For uniform mass and consistent API content, it is essential to ensure both homogeneous powder layer distribution and uniform binder deposition across the powder bed. Improper object orientation during the printing process leads to uneven powder spreading or incomplete wetting. As a result, mechanical properties and structural integrity of the final product could be compromised [73].

Despite the commercial approval of Spritam^®^, publicly available information regarding the implementation of PAT strategies during commercial-scale Binder Jetting manufacture remains limited. Most published studies focus on laboratory-scale process monitoring and quality assessment, while detailed post-approval PAT frameworks for the Aprecia manufacturing platform have not been extensively disclosed [73].

### 2.3. Laser-Based Technologies

#### Selective Laser Sintering (SLS)

Selective Laser Sintering (SLS) belongs to the laser-based 3DP techniques, where the powder mixture is solidified on a layer-by-layer basis due to the laser beam that draws a specific pattern on the surface of the powder bed. For this process to occur, an interaction between the laser beam and the powder particles (e.g., resin-based materials) is required [14].

The particle size and morphology are very important parameters for SLS 3DP, affecting the uniform sintering percentage (as shown in Table 3) [105]. Similar to other powder-based printing techniques, powder flowability is crucial, as a mixture with poor flowability creates unevenly distributed powder layers with agglomerated particles via Van der Waals intermolecular forces. To some extent, lubricants and glidants may help overcome flow-related issues [105,106].

Due to the use of a high-energy laser in the sintering process, API or excipients may undergo degradation, affecting the content uniformity of printlets. Although too strong laser power can degrade some of the UV-sensitive APIs, low radiation could result in poor printing resolution, as the resin may not cure properly, impacting the mechanical properties of the printlet [44]. Light-absorbing polymers could be a possible solution to overcome this aspect [14].

Laser scanning speed is directly correlated with printlet density (as shown in Table 3). Increasing the scanning speed results in lower printlet density and porosity. Hence, this CPP impacts the drug dissolution rate [107,108].

Powder bed temperature is a CPP controlled through the adjustment of the build platform temperature, and it is usually set below the Tg of the amorphous polymers, contributing to lower laser energy needed. However, some of the mixture ingredients may degrade, or the API could transform into a crystalline state, affecting the dissolution rate and the mechanical properties of the dosage forms, as the transition of the API from amorphous to crystalline form contributes to a lower packing density [75].

Layer thickness highly affects the dimensional accuracy and mass uniformity of the final dosage form. Thinner layers lead to a higher printer resolution, while thicker layers determine poor printlet resolution (as shown in Table 3). Therefore, the set layer thickness needs to be thinner than the average particle size of the powder. In some cases, thicker powder layers must be sintered, which requires higher laser energy and may lead to material degradation and altered mechanical properties of the printlets [16].

### 2.4. Light-Based Technologies

#### Stereolithography (SLA)

Stereolithography (SLA) is based on the ultraviolet (UV) light polymerization of photo-curable resins [14]. The liquid resin is mixed with the API in the form of a solution or suspension, and UV light will solidify the blend.

The key CMAs include the UV curability of the resin in the presence of a photo-initiator and its ability to maintain adequate stability.

Similarly to SLS, layer thickness affects the dimensional accuracy and mechanical properties of the printlets. It is inversely proportional to the tensile strength, as a decrease in layer thickness increases tensile strength, improving the dimensional accuracy, mechanical properties and the printing resolution as well (as shown in Table 3). Additionally, a thinner layer requires a smaller amount of laser energy, reducing the risk of material degradation. However, thinner layers increase the number of depositions [16,47,109].

Printing orientations impact the curing rate of the materials. Printlets with lower thickness should be vertically printed, as the horizontal printing orientation may not provide enough layers to achieve the level of overcuring needed for high-precision printing [109].

Curing parameters are important in SLA [109]. Laser power ensures proper curing of the material, and it can impact the mechanical properties and dimensional accuracy of the printlets. The higher the laser power, the higher the mechanical strength of the printlet and the higher the material shrinkage can be. Although a higher laser power can induce material degradation, an excessively low laser energy results in ineffective material curing, affecting printlet mass and dimensional accuracy (as shown in Table 3) [47,109].

Scanning speed is directly correlated with the laser power, as these two parameters define the laser energy exposure time of the probe, affecting the mechanical properties and dimensional accuracy (as shown in Table 3). A low-intensity laser combined with a high scanning speed results in incomplete material curing. However, fine-tuning of the laser energy and laser speed may be able to control these influences [47,109].

Hatch spacing is a unique CPP that represents the distance between the parallel vectors that are used to hatch the interior of the dosage form. Smaller hatch spacing causes inadequate laser exposure, affecting the dimensional accuracy. However, a higher hatch spacing leads to the entrapment of liquid resin into the object, affecting the mechanical properties of the printed tablets [47]. Hatch overcure represents the depth at which the vector penetrates, and it is a means to evaluate the adherence level of the new layer to the previous one. A smaller hatch overcure leads to a reduced laser exposure and a lower risk of shape and dimension change (as shown in Table 3) [16,47].

Overall, the CPPs identified for each printing technology demonstrate a direct impact on the CQAs of the printed product. Although CPPs highly depend on the printing technology, common trends can be observed, particularly their influence on content uniformity and mechanical properties.

**Figure 1:**
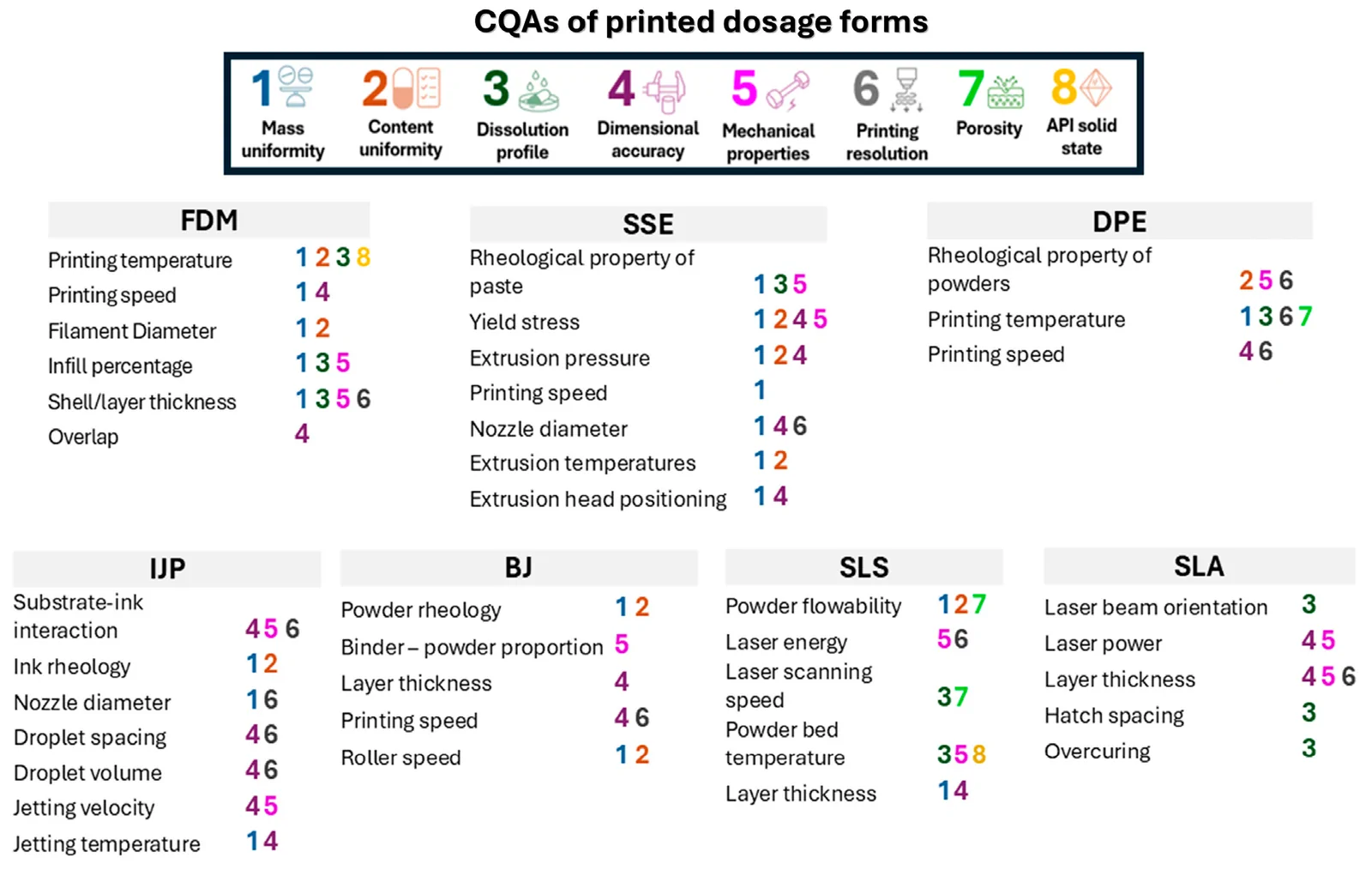
Main CPPs and CMAs involved in pharmaceutical 3DP and the CQAs they affect.

Importantly, the strong interdependence between CPPs and final product quality highlights the necessity of implementing robust monitoring and control, aligned with QbD principles. Figure 1 synthesises the main CPPs and the CQAs they affect, to have a broader overview of the process parameters in extrusion-, jet-, laser-, and light-based printing technologies, currently used in pharmaceutical 3DP.

**Table 3:** Main CQAs, CPPs and CMAs in IJP, BJ, SLS and SLA and their assessment through traditional vs PAT methods.

	CQAs	CMAs ^*^/CPPs	Conventional Methods	PAT Testing Methods	References
**IJP**	Mass uniformity	Jetting temperature ^*^ Ink rheology	Gravimetric measurement	-	[95,96,110,111,112]
	Content uniformity	^*^ Ink rheology	HPLC UV–Vis spectroscopy LC-MS NMR Confocal scanning laser microscopy	Transmission and reflection NIRS [113] Transmission/backscatter Raman [113] Raman chemical imaging [100] Colorimetry [114]	
	Dissolution profile	Printing temperature	Disintegration tester In vitro dissolution tester	-	
	Dimensional accuracy	Jetting temperature Jetting velocity Droplet volume Droplet spacing Substrate–ink interaction	Surface thickness measurement (micrometre device)	-	
	Mechanical properties	Jetting velocity Substrate–ink interaction	Texture analyzer Air permeation tester Friability tester	-	
	Printing resolution	Droplet volume Droplet spacing Substrate–ink interaction	Polarised light microscopy SEM SWLI EDX Optical microscopy	Raman chemical imaging [1]	
	Porosity	Substrate–ink interaction	ATR-FTIR DSC	-	
**BJ**	Mass uniformity	^*^ Powder/binder rheology Roller speed	Gravimetric measurement	-	[44,73,115]
	Content uniformity	^*^ Powder/binder rheology Roller speed	HPLC UV–Vis spectroscopy	-	
	Dissolution profile	Printing temperature	In vitro dissolution tester	-	
	Dimensional accuracy	Printing speed	Diameter measurement (digital caliper)	-	
	Mechanical properties	Powder layer: binder proportion	Texture analyzer	-	
	Printing resolution	Printing speed	Polarized light microscopy	-	
	Porosity	Printing speed	SEM	-	
**SLS**	Mass uniformity	^*^ Powder rheological properties Powder layer thickness	Gravimetric measurement	-	[44,68,105,108]
	Content uniformity	^*^ Powder rheological properties	HPLC UV–Vis spectroscopy	NIR reflectance Raman spectroscopy [105]	
	Dissolution profile	Laser scanning speed Solid state of API	In vitro dissolution tester	NIR reflectance [108] NIR reflectance and Raman spectroscopy [105]	
	Dimensional accuracy	Powder layer thickness	Diameter measurement (digital caliper)	-	
	Mechanical properties	Laser power Powder bed temperature	Texture analyzer	-	
	Printing resolution	Laser power	SEM	-	
	Porosity	Laser scanning speed	XRPD DSC	-	
**SLA**	Content uniformity	Powder layer thickness ^*^ Rheological properties of powder	HPLC UV–Vis spectroscopy	Luminescence-labelled surface mapping system [116] Raman spectroscopy and mapping [117]	[116,117]
	Dissolution profile	Laser power	In vitro dissolution tester	-	
	Dimensional accuracy	Laser power Laser scanning speed Powder layer thickness	Diameter measurement (digital caliper)	-	
	Mechanical properties	Laser power Laser scanning speed Powder layer thickness	Texture analyzer	-	
	Printing resolution	Powder layer thickness	SEM	-	

* = CMAs; ATR-FTIR = Attenuated Total Reflectance–Fourier-Transform; BJ = Binder Jetting; DSC = Differential Scanning Calorimetry; EDX = Energy-Dispersive X-Ray Spectroscopy; IR = Infrared; IJP = Inkjet Printing; LC-MS = liquid chromatography–mass spectrometry; NMR = nuclear magnetic resonance; SEM = Scanning Electron Microscopy; SLA = Stereolithography; SLS = Selective Laser Sintering; SWLI = Scanning White Light Interferometry; XRPD = X-Ray Powder Diffraction.

## 3. Process Analytical Technology in Point-of-Care Manufacturing

According to the U.S FDA, PAT is an approach used to design, monitor, and control manufacturing processes through real-time assessment of CQAs, with the aim of ensuring consistent final product quality [10].

The constant development in technology, software, design, and instrumentation has led to non-invasive techniques for the quality control of pharmaceutical dosage forms, such as NIRS and Raman spectroscopy, X-ray microtomography, nuclear magnetic resonance (NMR) imaging, terahertz pulsed imaging, laser-induced breakdown spectroscopy, and several thermal or acoustic-based control methods [118,119,120]. XRPD and Micro-Computed Tomography (µCT) are also non-destructive PAT tools for physical form analysis, but due to their long time span and requirement for expensive equipment, the currently existing studies using such technologies are limited [105,108].

These non-destructive QC tools can be used to monitor the CQAs of printlets, such as drug content, mass uniformity and dissolution profile [18]. The most widely studied PAT tools in the pharmaceutical QC of printlets will be discussed below.

### 3.1. Near-Infrared Spectroscopy (NIRS)

NIRS is a type of low-energy vibrational spectroscopy, covering the electromagnetic spectrum between 780 and 2500 nm, where the fundamental vibrations and overtones of the functional groups (-NH; -CH; -OH; -SH) present in the molecules of the sample interact with radiation, resulting in specific spectral bands due to a dipole moment change in the molecule [121,122]. NIRS in reflectance, diffuse reflectance, and transmittance is mostly used in pharmaceutics as a PAT tool [11,123,124,125].

Since NIR spectra contain many overlapping overtones and are hard to interpret, they require spectral preprocessing, called chemometrics, that can extract the relevant information [126]. The mathematical pre-treatment, such as Standard Normal Variate (SNV), Multiplicative Scatter Correction (MSC), and derivates are meant to reduce background information, minimise the errors caused by light scattering, and increase the signal of the analysed chemical compound [2,86,127,128]. Smoothing factors, such as Savitzky–Golay, can also be applied to reduce the spectral noise [128].

As light energy is low in NIRS, the chances of dosage form damage are very low [121]. In NIRS, samples need very little or no preparation prior to analysis, which is crucial for the QC of printlets in clinical settings or pharmacies, as they can be analysed in their original form [129].

NIRS measuring technique will depend on the characteristics of the dosage form and optical properties of the samples, such as their scattering properties. For instance, transparent samples are mostly measured in transmittance [128]. Optimal sample presentation is highly important, especially when measuring solid samples, as variations in packing density may induce stray light or scattering effects, causing errors in the spectra [128,130,131]. One of the main sources of spectral error in NIRS is improper sample positioning, particularly for solid samples, due to variations in scattering effects and the presence of stray light when samples are not positioned correctly. This is why appropriate sample holders are of utmost importance in NIRS [128]. Moreover, NIRS is highly influenced by the humidity of the samples and the humidity and temperature at the point of testing [119], so a lack of proper control over humidity and temperature can lead to substantial errors, as reported by Yang et al., where the linearity of the calibration model was affected [86,128].

NIRS-reflectance provides only a surface analysis of the dosage form, having a penetration depth of only several millimetres (1–3 millimetres) [7,129]. Hence, the use of NIRS-reflectance for in-depth analysis of samples could only be implemented in-line, on a layer-by-layer analysis, to provide an overall view of the printlets [7]. On the other hand, transmittance spectra could provide a better overview of the scanned dosage form, as they penetrate through the sample, but they also have their limitations, as a substantially thick sample can have minimal to no signal [7,128]. Both techniques require a minimal sample volume of 75 mm^3,^ 6.5 times larger than Raman spectroscopy needs.

Spectral data can be exploited to characterise API and polymer composition, assess moisture levels, and support indirect dissolution prediction using Multivariate Data Analysis (MVDA). This spectroscopic PAT tool can be implemented at-line, on-line or in-line into the process to provide real-time process information [128]. Thanks to the miniaturisation of these tools by creating portable, handheld devices, in situ analysis is possible [129].

This PAT tool does not require major sample processing and specialised personnel, and the costs of this method are significantly reduced compared to QC through traditional testing methods [132]. However, the requirement for multivariate calibration development can be a drawback, as the process can be time-consuming and require good knowledge of MVDA [133]. Several studies proved the feasibility of NIRS to assess the CQAs in 3DP technologies, which will be later described as examples.

### 3.2. Raman Spectroscopy

Raman spectroscopy is a vibrational technique that measures the inelastic scattering of light caused by interactions with the molecular electron cloud, revealing changes in the polarizability of molecular bonds [2,134]. In Raman scattering, the incident light briefly excites the molecules to a virtual state; as they relax back, the resulting energy difference causes a Stokes or anti-Stokes wavelength shift [2,118]. Raman spectroscopy may be configured in transmittance or backscattering modes [7].

Similarly to NIRS, Raman spectra need to be preprocessed using chemometrics to extract the valuable information [135]. The most common preprocessing technique for baseline correction is Asymmetric Least Squares Smoothing (AsLs), but further noise reduction could be achieved by first or second derivatives, as well as Savitzky–Golay smoothing filters [2].

Raman spectroscopy has higher light intensity than NIRS, which could damage thermolabile ingredients in dosage forms. Moreover, this technique may have a lower quantification sensitivity, and it is significantly affected by the effect of fluorescence, as fluorescence could completely obscure the spectra and prevent the extraction of relevant information [2,129]. To reduce these negative effects and to have a good signal intensity, most devices use a 785 nm laser for excitation [129].

In Raman spectroscopy, each material has its characteristic shift that depends upon its chemical composition. The width of the Raman peak can assess sample quality, while the peak intensity provides quantitative information about the sample [118,121]. Despite identical chemical composition, the Raman spectra of different solid-state forms of a drug can vary due to differences in molecular packing and long-range translational symmetry present in crystalline phases but absent in amorphous forms [105]. As Raman spectra are characterised by high dimensionality, they can be combined with machine learning to extract the essential chemical information and to apply this tool in the QC of 3D-printed products [136].

This PAT instrument is cost-efficient and requires minimal to no sample preparation [136]. Raman spectroscopy has a penetration depth of just several millimetres and necessitates a small spot size for analysis (1–2 mm); thus, there is a need for a minimal sample volume of 10 mm^3^ [129]. A variety of information can be extracted from spectral data, including API identification and assessment, as well as the solid-state form of API and excipients [137]. This PAT tool usually may take longer than NIRS analysis, but it is minimally affected by the interference of water, as Raman spectroscopy is a weak water scatterer [138,139].

It can be easily implemented on-line and in-line into the monitoring of CQAs of dosage forms, but also for the QC of the intermediate product [122,140].

### 3.3. Attenuated Total Reflection–Fourier Transform Infrared Spectroscopy

While Infrared (IR) spectroscopy is primarily used to identify chemical entities and polymorphs, there are emerging examples of its integration as a PAT tool in pharmaceutical manufacturing technologies.

IR spectroscopy, like NIRS, analyses the molecular structure of samples by providing information on intramolecular vibrations, but in the mid-infrared region. Absorption of IR radiation occurs when a change in the molecule’s dipole moment happens [129]. Attenuated Total Reflection–Fourier Transform Infrared (ATR-FTIR) spectroscopy enables the acquisition of IR spectra without the need for sample pretreatment, allowing it to be effectively used for surface analysis. ATR-FTIR is a sensitive technique for surface analysis, particularly useful for characterising challenging samples, detecting solid-state transitions, and quantifying polymorphic content in pharmaceutical dosage forms. It is especially effective for identifying homogeneous compounds present at low concentrations [141].

Like NIRS and Raman spectroscopy, there is a need for spectral preprocessing to extract the relevant information and to build the proper model based on spectra. In the current literature, SNV has been predominantly used as a spectral filter to reduce baseline drifts in the spectra [142].

Sample properties of ATR-FTIR spectra quality are highly affected by the presence of heterogeneous particles (ex., polymorphs) in the sample, which can cause signal overlaps, baseline drifts, and light scattering and can result in the obstruction of the relevant spectral information [136].

This technology provides only a surface analysis, having a low penetration depth [136]. Spectral information may be primarily used for chemical identification, details regarding API–excipient interaction, and information regarding chemical degradation [141].

The main limitation of this PAT tool lies in its inability to be implemented in-line, which explains the limited number of studies regarding this technique [142]. Table 4 compares the spectroscopic PAT technologies with respect to their penetration depth, sample properties, and their in-line implementation in the QC of the technological process.

### 3.4. Hyperspectral Imaging

Hyperspectral imaging is an innovative PAT tool; the main advantage over other spectroscopic techniques lies in the additional spatial dimension provided by the addition of an imaging technology, which enables the generation of chemical images. These images allow not only the identification but also the spatial localization of the chemical compounds within non-homogeneous samples. Hyperspectral imaging techniques, such as NIRS, Raman, and mid-infrared imaging, were found to provide a vast amount of spectral and visual information from the sample surface analysis [143]. Recent instrumentation advancements have led to the rapid analysis of tablets and 3D-printed dosage forms via these techniques. Currently, studies are focused on NIRS and Raman spectroscopy imaging.

#### 3.4.1. NIRS Hyperspectral Imaging (NIRS-HIS)

A more advanced PAT tool for both qualitative and quantitative analysis could be the combination of NIRS with modern imaging technology, where 3D data can be created, combining the spatial and spectral information represented on the three axes [118]. The sample is irradiated with NIRS light, and the diffuse reflectance image of the sample is then collected, obtaining micro/macroscopic images, according to the experimental requirements. A series of images is then recorded, and the results are represented in the form of a spectral hypercube, where x and y represent the spatial information and z represents the spectral information acquired [127,138]. One measurement can collect tens of thousands of spectra, where each spectrum reflects a certain area (one pixel) on the surface of the sample. By collecting all the spectra into one image, highly precise information regarding the API distribution at each point on the surface of the sample can be verified [48]. Thus, NIRS imaging is a stronger tool than NIRS, providing not only physico-chemical properties of the samples, but also the spatial distribution of the components, which is a major advantage for heterogeneity checking [2,128].

In some cases, pure component spectra can be extracted from the spectral image using Multivariate Curve Resolution (MCR) techniques. The important spectral information extracted serves for the qualitative and semi-quantitative assessment of the dosage forms. Moreover, it reduces both time and costs, particularly when working with expensive drug formulations [137].

Similarly to NIRS, it can be applied for on-line and in-line QC of medicine [49].

#### 3.4.2. Raman Chemical Imaging

Like NIRS chemical imaging, Raman spectroscopy is often integrated within an optical microscope to enable high-resolution chemical mapping of sample composition [137]. This analysis method collects a high number of spectra over the analysed area and creates complex image data that can provide information regarding drug distribution and drug load. Due to imaging techniques, drug distribution becomes more visible and understandable [2,144]. Colour-coded mapping profiles are created, where the various signal intensities are grouped and presented in several colours, creating an observable map, where drug or other excipient distribution can be easily observed [98].

A comparative study between Raman and NIRS imaging technologies affirmed the superiority of Raman chemical imaging, because the Raman image revealed sharper and better discriminated domains for each component, having a higher spatial resolution, whereas large, pixelated regions dominated NIRS chemical imaging [145].

Similarly to Raman spectroscopy, on-line and in-line implementation is possible in the case of Raman chemical imaging and will be later exemplified [146].

### 3.5. Colorimetry

Colorimetry relies on the indirect API quantification based on the colour intensity of the printlets. It is mainly used in IJP. Since most APIs lack colours, a colouring agent needs to be added to the ink formulation [2].

Unlike spectroscopic PAT tools, this quantitative method does not require the use of chemometrics [111].

The use of colorimetry requires the API to be freely soluble in the solvent, insofar as an ink containing precipitated API could lead to an improper drug content determination [114]. Furthermore, the absorption capacity of the substrate could reduce the colour intensity, also leading to inaccurate API quantification. Therefore, the absorbing capacity of substrates needs to be evaluated first [2]. Another limitation of colorimetry could be the risk of colour saturation. To avoid this, the incorporation of colouring agents should be optimised with respect to the intended dose range, and in some cases may require increasing the dosage form diameter [111].

Although colorimetry proved to be a suitable method for the on-line QC of dosage forms, reliable prediction of drug load requires establishing a strong correlation between ink colour intensity and API content, as well as selecting an appropriate substrate [2].

### 3.6. Terahertz Pulsed Spectroscopy and Imaging

Terahertz spectroscopy is an absorption-based technique that covers the spectral range between mid-infrared and microwave radiation. This region can be named far-infrared radiation, and it is dominated by intermolecular vibrations, which correspond to the delocalized movements of a vast number of molecules and atoms. This technology differentiates identical molecular structures that have different crystal forms [122]. When combined with an imaging technique, it can show the microstructure of the samples [6].

Compared to other spectroscopic PAT technologies, such as NIRS or Raman spectroscopy, there is no need for complex calibration models and use of chemometrics prior to analysis.

It has a penetration depth of 5.3 mm, and water content or crystallinity can significantly impact the terahertz wave propagation through the sample, affecting the results [136]. It is a non-invasive tool for QC; it can provide an in-depth analysis below the sample’s surface, evaluating the solid state of the API inside the printlets (detection of polymorphs, the analysis of crystal structures), as well as their porosity [122,136,147,148].

It is mostly used in the evaluation of film-coated dosage forms in traditional manufacturing, but it could be implemented and studied in the 3D printlet property evaluation too [122,147,148]. A possible method could be coupling with NIRS to obtain complementary data of the samples analysed [136].

There are few research articles that have evaluated the use of terahertz spectroscopy in 3DP (SLS 3DP), proving to be a meaningful characterisation tool for solid-state analysis of samples, including printlets [45].

This PAT tool is currently implemented only in an on-line manner, due to its high complexity [136].

### 3.7. Acoustic Emission Technology

Acoustic emission technology (AET) measures the acoustic vibrations that are generated by particle-particle or particle-chamber collisions and frictions during the process. These waves are captured by a piezoelectric detector and are further converted into electrical signals [136]. There is a variation in the acoustic signal when particle characteristics change over time. Using MVDA, these signals can be further processed to detect plastic deformation, friction and phase transformations [81,122,149].

It has been implemented in-line in the powder flow monitoring during the solid oral dosage form production and the determination of API content during the tabletting process. Since AET is very sensitive to environmental noise, such as particle collision and mechanical vibrations, it is not the best tool for CQA monitoring [136,150].

## 4. Process Analytical Technology in 3DP Process Monitoring and Quality Control

Most of the currently existing research articles on 3D-printed drugs use conventional QC methods to characterise the dosage forms, such as high-performance liquid chromatography (HPLC), gas chromatography, liquid chromatography–mass spectrometry, or UV–Vis spectroscopy, instrumental texture analysis, and in vitro dissolution testing. These methods are also used in the industry as standard QC tools, according to the Pharmacopoeia [151]. However, they are destructive, time- and money-consuming methods that require highly skilled operators, which could limit their implementation at the PoC [1,7,25,36,61,107,152]. Unlike batch manufacturing, where destructive QC methods can be applied to representative samples, 3DP produces unique, patient-specific units that cannot be sacrificed for testing. This makes non-destructive QC essential to eliminate redundant production, reduce costs, and enable the real-time release testing that PoC 3DP requires.

### 4.1. Extrusion-Based Technologies

#### 4.1.1. Fused Deposition Modelling

To guarantee that the CQAs are within the desired limits, the integration of analytical techniques into the process is highly important [2,6]. Several attempts have been made to implement PAT tools (NIRS and Raman spectroscopy and imaging) for the main CQAs monitoring of FDM printlets and HME filaments, mainly drug content, dissolution rate, and mechanical properties.

Saerens and colleagues were among the first to implement an in-line PAT tool in the HME process, developing and validating a Raman-based Partial Least Squares (PLS) regression model for the quantification of metoprolol tartrate in the hot-melt extruded filaments, using a Raman probe inserted into the twin-screw extruder die [80]. Beyond quantification, in-line Raman spectroscopy allowed the monitoring of the polymer–API solid-state at two processing temperatures, where broadened peaks indicated an amorphous solid solution and sharper peaks corresponded to a crystalline solid dispersion [80]. A subsequent study from the same group highlighted that Root Mean Square Error of Prediction (RMSEP) alone is insufficient for robust model validation, recommending the additional use of β-expectation tolerance intervals [153]. Using a similar in-line collected Raman spectra database, both PLS and Multivariate Curve Resolution (MCR) models were developed for metoprolol tartrate quantification in extruded filaments. The MCR model yielded superior predictive performance, with only one concentration falling outside the β-expectation tolerance interval, attributed to an unexplained elevated background signal on day 1. Given that validation was conducted across three days and multiple operators, this variability may reflect an operator effect, potentially related to inconsistent fixation of the Raman probe within the extrusion die. Furthermore, the impact of extrusion temperature on the API solid state was assessed through Raman spectral peak shift analysis, demonstrating that processing above the Tg of the API–polymer mixture resulted in complete API amorphization, confirming that extrusion temperature is a critical parameter governing the solid-state properties of the API in the filaments [153].

While API quantification through PAT tools showed positive results, the risk of API degradation during HME requires simultaneous monitoring of byproduct formation. Andrews et al. investigated in-line Raman spectroscopy for the detection of ramipril and its degradation byproducts in extruded filaments [154]. Using Principal Component Analysis (PCA) mapping of the in-line collected Raman spectra, both API levels and degradation byproducts could be simultaneously detected, demonstrating the potential of PAT tools for real-time degradation monitoring. Additionally, feeding speed and screw speed were identified as CPPs influencing byproduct formation and content uniformity, though their relationship was not systematically explored through a full experimental design. A limitation of this study was the absence of PLS model validation, despite successful identification of spectral modifications associated with API and byproduct changes [154]. Islam et al. further investigated the influence of screw speed on both product quality and spectral performance, implementing in-line transmission and reflectance NIR probes simultaneously into a twin-screw extruder to monitor the solid-state transformation of indomethacin during HME [155]. The transmission probe was positioned at the extrusion die, while the reflectance probe was placed at the first mixing zone, enabling monitoring of the API crystalline-to-amorphous transformation along the barrel. PCA demonstrated that increasing screw speed significantly affected spectral repeatability due to concurrent increases in die pressure and torque, confirming that process parameters must be kept constant for reliable NIR monitoring. However, no PLS model was developed or validated for quantitative API determination, limiting the approach to qualitative solid-state monitoring. Additionally, the reflectance probe proved unsuitable for transparent extrudates due to excessive noise, restricting its applicability in molecularly dispersed HME systems [155]. Although the CPPs governing filament quality during HME are well defined, the application of PAT tools in HME remains limited, with existing studies applying spectroscopic techniques mostly individually and for one CPP/CQA.

As an innovative approach, Dowek et al. built the calibration model on a hydrochlorothiazide-containing solution by Raman spectroscopy and transferred the PLS model directly to solid filament spectra [156]. A band shift at 1611 cm^−1^, arising from the liquid-to-solid phase transition, required explicit correction prior to prediction. Selection of an excipient-free spectral window (1552–1636 cm^−1^) combined with extended multiplicative signal correction preprocessing allowed cross-matrix application without rebuilding the calibration [156]. Future work should focus on extending the research on calibration transfer and the integration of multiple PAT instruments simultaneously throughout the HME process to generate a complete real-time quality profile of the filament, prior to its use as feedstock for 3D printing.

The number of PAT studies applied to FDM 3DP quality control remains limited due to challenges associated with PAT sensitivity and process-related limitations of FDM. An important issue was highlighted by Yang et al., who studied the applicability of NIRS in the caffeine content assessment of printlets produced by FDM, during and after printing [152]. The model built for the characterisation of the final dosage forms could not be used for the API assessment during the printing process. Different infill patterns and levels of outer shell completion resulted in variations in surface morphology, which in turn influenced the NIR diffuse reflectance spectra. Therefore, a model was built for each completion stage (20–40–60–80%), so the drug content could be effectively predicted in both cases. The multiple stacked layers of the printlets with inter-layer seem line contribute to the spectral data variability, especially in the case of NIR diffuse reflectance. A linearity of the PLS regression model of 0.985 has been achieved for the full completion model, which, according to the authors, could have been improved by tight control of humidity and unit positioning during the NIRS scan. Moreover, in the validation process, the low API-content printlets had an RSD > 2%, which might suggest that NIRS has a reduced sensitivity for smaller API concentrations [86]. Another challenge when implementing NIR sensors into the printing process could be the difference between printing speed and NIR scan time. In most cases, it would require an interruption of the 3D printing process, which could affect the final product quality. One possible solution could be shortening the scan time and narrowing the inspected wavelength range [86].

The low sensitivity of NIRS for lower concentrations was sustained in another study by Yang et al., where in-line NIRS was used to assess drug content in the FDM printlet containing a thermolabile API, hydrocortisone. The printing temperature was taken into consideration as CPP, influencing the drug content of the printed dosage forms. As in the examples mentioned above, a PLS regression model was built as the calibration model used for prediction of API content in future printlets. The model was able to predict the API concentration in a wide range, between 0 and 15%, which is an important aspect, as in many cases low API concentration cannot be assessed by NIRS calibration models. A high linearity (R^2^ = 0.981) and accuracy (Root Mean Square Error of Cross-Validation = RMSECV) of 0.47% characterised the model, proving the possibility to develop calibration models that detect API concentration in a wide range through NIRS. As described in the first chapter, the printing temperature and polymer–API ratio were key factors in attaining acceptable drug content levels, confirming the critical role of CPPs in achieving the desired QTPP [152]. Although external validation was performed through comparison with a reference method and supported by low prediction bias and acceptable repeatability, RMSEP was not explicitly reported. Nevertheless, the combination of bias, RSD, and agreement with reference values indicates satisfactory predictive performance of the NIR model [152].

The same low sensitivity and low API concentration were reported by Khorasani et al., who applied NIRS chemical imaging in the off-line drug content and drug distribution analysis of FDM printed solid dosage forms containing nitrofurantoin [157]. The spectral and image information were analysed using Multivariate Curve Resolution–Alternating Least Squares (MCR-ALS), a semi-quantitative method. Based on the image information, drug distribution could be visualised within the surface of the solid dosage form, and also the API content could be estimated, highlighting the applicability of NIRS-CI for the QC of FDM printed dosage forms [157]. Important to note that this method has not been validated, so further validation would ensure proper API quantification results. During HME, nitrofurantoin monohydrate suffers a dehydration process, which could be observed by NIR-CI with MCR-ALS.

When assessing API content using PAT tools, a major limitation of the FDM process is the long printlet production time, mainly associated with the HME step. As an innovative approach, Jørgensen et al. assessed the API content of VCM-prepared formulations loaded with 10–30% tamoxifen as FDM printlet surrogates through NIRS [5]. The calibration model presented low calibration (RMSEC = 0.19%) and prediction errors (RMSEP = 0.20%) for tamoxifen. The model was capable of accurately predicting the API concentration (R^2^ = 0.997, Q^2^ = 0.996), and results were confirmed by the HPLC content analysis of the dosage forms [5]. This is the first study to present a calibration transfer in FDM 3DP from VCM tablets to FDM printlets, drastically reducing the fabrication time for developing and validating the regression model.

The above-mentioned studies only evaluated the API content and/or API solid state; however, there are many CQAs that still have not been studied in FDM. Dissolution has been investigated by Whalley et al., using NIR and Raman mapping [59]. Two distinct geometries were analysed, a solid tablet and a channelled one, to observe the differences in water ingress and the component changes throughout dissolution. By the use of NIR and Raman mapping, as high-resolution techniques, the dynamic dissolution process and the rate of water ingress were observed, and as expected, the channel additions into the formulations led to faster API release [59]. However, the aim of the study was only to demonstrate the possibility of using chemical mapping in dissolution observation, and the need for more in-depth understanding is of utmost importance.

Despite NIR and Raman spectroscopy, other PAT tools such as optical coherence tomography, X-ray microtomography, and terahertz pulsed imaging (TPI) represent potentially applicable alternatives for non-destructive printlet characterisation, though published data on these techniques remains scarce even in conventional tablet QC contexts. Markl et al. demonstrated that μ-CT and TPI could characterise FDM printlet microstructure and correlate porosity with dissolution behaviour, with Polyvinyl alcohol and Polylactic acid producing markedly different pore architectures of 5.5% and 0.2% porosity, directly influencing drug release kinetics [85]. Neither technique had been adopted in subsequent 3DP QC studies. μ-CT acquisition and reconstruction exceed one hour per sample, making individual unit control incompatible with any Point-of-Care workflow, while TPI, though considerably faster, did not permit quantitative porosity determination. Critically, neither technique addresses API content directly, limiting their practical utility relative to spectroscopic PAT tools capable of non-destructive drug quantification within seconds [85]. Despite spectroscopic PAT tools, the feasibility of several sensor implementations into FDM has been researched [68,82]. Soriano et al. developed a custom-designed sensor attached to the FDM 3D printer to detect the filament advancement in the 3D printer during the process, using a rotation encoder driven by the movement of the filament. The parameters from the sensor transmitted to the control software could precisely detect the filament advancement level. If no advancement was detected, the equipment would automatically stop, allowing operator intervention to solve the issue [82]. Yang et al. were the first to employ an acoustic emission sensor to monitor filament breakage within an FDM 3D printer [81]. Monitoring filament advancement therefore offers valuable insight into process performance, enabling the early detection of feeding irregularities and supporting timely feedback for improved process control. Further studies can be done to monitor other parameters in the FDM or other pharmaceutical 3DP processes [81].

#### 4.1.2. Semisolid Extrusion

Although SSE is a well-established and increasingly adopted 3DP platform for PoC manufacturing, the number of studies incorporating PAT-based QC (particularly NIRS or Raman spectroscopy) remains limited. This likely reflects the absence of standardised integration strategies for spectroscopic monitoring in SSE systems, and practical instrumentation constraints that complicate the incorporation of robust in-process analytical tools. Shokraneh et al. integrated for the first time at-line NIRS for real-time SSE paste blend monitoring alongside on-line mass-uniformity testing, enabling early detection of pre-printing formulation inconsistencies [94]. However, NIRS was limited to at-line blend assessment rather than in-process printlet control, and despite acceptable PLS performance (R^2^ = 0.91; RMSECV = 0.38), the narrow calibration range (1–2% *w*/*w* furosemide) and small dataset restrict model robustness. Mass-uniformity reporting is incomplete, as only Ph. Eur. compliance is stated without underlying mass-variation data, RSD values, or individual printlet weights [94]. Further studies have concentrated on implementing in-line temperature and pressure sensors and weight measuring systems. These systems allow the determination of any irregularities related to material extrusion and mass variation during SSE 3DP [40,158,159].

A significant gap remains in the application of spectroscopic PAT tools within SSE-based 3DP, and addressing this gap represents an important research direction for advancing in-process control and regulatory-aligned quality assurance.

#### 4.1.3. Direct Powder Extrusion

Similarly to SSE 3DP, there is a limited number of publications regarding the use of spectroscopic PAT tools in DPE 3DP.

Mora-Castano et al. demonstrated the feasibility of integrating an in-line analytical balance for weight uniformity measurement in DPE printing, where process conditions are considerably more challenging than in SSE, due to elevated temperatures, vibrations, and smaller layer heights causing frequent contact with the balance platform [95]. To ensure reliable mass determination, the cooling fan was disabled during measurement and the printhead physically isolated to prevent airflow interference. While this represents a significant step forward, monitoring of additional critical process parameters and quality attributes remains a priority [95]. The first in-line NIRS implementation for API quantification in DPE printlets was reported by Seoane-Viano et al. The study served as a proof-of-concept for integrating spectroscopic PAT into DPE: a portable MicroNIR unit was mounted on the printer’s moving component, and using specialised software, the system repositioned after each print to allow the attached probe to scan every individual printlet. However, the chemometric model was not subjected to any formal accuracy, precision, or specificity assessment, leaving its analytical performance uncharacterised [38]. API quantification was further assessed by Jørgensen et al., who demonstrated and accurately validated the feasibility of assessing printlet drug loading using transmission Raman spectroscopy as a PAT approach [88]. Unlike reflectance-based methods previously explored by Seoane-Viaño et al., samples were individually transferred into Transmission Raman trays, introducing a manual handling step and a potential bottleneck in a decentralised manufacturing workflow. Although the PLSR model showed strong predictive performance validated against HPLC, it was built using a single pharma-ink with only printlet size varying and surface area-to-volume ratio kept constant, raising concerns about whether predictive ability would be maintained upon changes in this ratio—a more clinically relevant scenario for personalised medicine production [88]. However, there is a need for further studies implementing spectroscopic PAT tools in the QC of printlets produced via DPE, which can also be seen in Table 2.

### 4.2. Jet-Based Technologies

#### Inkjet Printing

As IJP is an intensively studied 3DP technique in the pharmaceutical field, there are numerous studies that tried to apply PAT tools, such as NIRS, Raman spectroscopy or colorimetry, in the QC of IJP dosage forms.

Similarly to FDM and SSE 3DP, the API quantification had higher prediction errors for lower API concentration, due to the presence of background noise in the spectral signals. Vakili et al. integrated in-line NIRS in the API quantification of inkjet-printed formulations containing prednisolone and levothyroxine, on two different substrates [160]. The models were built on the analysis of 4 cm^2^ orodispersible films with different API concentrations. The predictability of the OPLS models developed for both APIs was between 0.941 and 0.988, and good to excellent linearities (R^2^ = 0.948-0.990) were obtained [160]. Furthermore, the authors affirmed that the models were built on analysing a relatively small area, which is not representative of the whole sample. If integrating PAT tools into 3DP techniques, a large quantity of spectral data needs to be acquired to have an overview of the whole printlet, especially for low-penetration PAT tools.

As a means to control droplet volume dispensing in IJP, Stranzinger et al. used NIR-HIS/PLS for quantitative assay and drug distribution mapping of inkjet-printed metformin hydrochloride on gelatine substrates, outperforming printer-derived volume data and correlating well with HPLC across 1–90 µg/mL. Raman imaging served solely to correct coffee-ring-induced calibration gradients, with no Raman vs NIR-HIS comparison reported. The model remains at the pre-validation stage, with multilayer signal attenuation, ink migration, non-investigated inter-layer drying, and absence of any CPP/CQA framework as key unresolved limitations [49].

Since IJP relies on the deposition of drug-containing inks onto carrier substrates, the contribution of substrate-related spectral signals remains a challenge for NIR-based analysis. This issue was highlighted by Vakili et al., who used NIRS-HIS to evaluate the distribution and content of theophylline printed onto commercial copy paper substrates [48]. Prediction errors increased at lower theophylline doses, which was attributed to the limited sensitivity of the NIRS method and the influence of substrate-derived spectral features on the acquired spectra [48]. However, a major limitation of this study was the use of commercial paper as the printing substrate. As paper is not suitable for pharmaceutical administration, the work primarily demonstrated the feasibility of the analytical approach rather than its direct applicability to clinically relevant dosage forms. Moreover, validation relied primarily on R^2^ values and visual scatter plots, providing only limited evidence of model robustness and predictive capability. A persistent gap in PAT application to Inkjet Printing is that substrate behaviour remains an uncontrolled CPP, yet it critically determines whether spectroscopic quantification is even feasible. In this study, Raman spectroscopy could quantify haloperidol on CaHPD compacts, but accuracy was compromised by ink penetration beyond the Raman sampling depth, by strong matrix interference on paracetamol tablets, and by complete signal loss on low-density Hydroxypropyl methylcellulose foam [146]. These results show that, alongside CPPs such as ink viscosity and surface tension, substrate selection becomes a prerequisite for PAT success, because a single spectroscopic tool cannot remain reliable across substrates with fundamentally different optical and structural properties [146].

Palo et al. evaluated ATR-FTIR spectroscopy combined with PLS regression as an at-line PAT approach for the quantification of printed caffeine and loperamide hydrochloride, with dose modulated via drop spacing (10–50 μm) on PET substrates [142]. The best PLS models, obtained using SNV preprocessing and mean centering in the 400–1750 cm^−1^ fingerprint region, yielded R^2^Y = 1.00, Q^2^ = 0.98, RMSEP = 0.22 log mg for caffeine and R^2^Y = 0.98, Q^2^ = 0.95, RMSEP = 0.16 log mg for loperamide, demonstrating acceptable predictive performance for both a crystalline and a non-crystalline printed API. Nevertheless, ATR-FTIR remains inherently limited to at-line application unlike Raman or NIRS, and further work should address API distribution heterogeneity, substrate and excipient spectral overlap in more complex formulations, the non-investigated effect of measurement pressure on solid-state transitions, and the absence of full ICH-compliant method validation [142]. Using the PLS regression model, both caffeine and loperamide hydrochloride concentration within printlets could be determined with good predictability [142]. Although ATR-FTIR has rarely been studied as a PAT tool in 3DP, because it could only be used at-line, it could be a promising tool for QC [142].

Edinger et al. developed and validated calibration models assessing the API content of inkjet-printed oral films, through NIR transmittance and Raman spectroscopy, as complementary PAT tools [113]. The study was conducted on three APIs, propranolol, montelukast and haloperidol. The PLS regression models for both NIRS and Raman spectroscopy had high linearities (R^2^ between 0.95 and 0.99), although the best predictive models were obtained by NIRS. NIRS outperformed Raman because the porous Hydroxypropyl methylcellulose substrate produced weak Raman scattering but allowed clean NIR transmission/surface signals, making NIRS less affected by substrate-dependent variability. The authors note that Raman performance was API-dependent (e.g., fluorescence from propranolol ink and weak Raman signal for haloperidol), whereas NIRS consistently produced stable, high-quality spectra across all three APIs [113]. Therefore, the use of various PAT tools depends on the properties of the API.

Colorimetry has been explored as a PAT approach for API quantification in IJP. Studies using propranolol and vitamin B inks showed that colour intensity could be correlated with API content, yet issues regarding smearing on low-permeability substrates and colour saturation after multiple layers highly compromised the feasibility of the method. Despite early proof-of-concept studies, colorimetry has not been adopted in recent 3DP research because the method’s linearity and predictive performance are highly formulation- and substrate-dependent [111].

### 4.3. Laser-Based Technologies

#### Selective Laser Sintering

In recent years, there has been an extensive application of PAT tools in the QC of SLS 3DP products, mainly of NIRS and Raman spectroscopy.

Most PAT studies assess API quantification models using a single dosage form design. However, despite the design flexibility offered by 3D printing, only a limited number of studies have investigated model transferability across different dosage form geometries. Trenfield et al. developed and validated a model that could predict paracetamol concentration in 3D printlets, produced via SLS, using various designs: cylindrical, torus-shaped printlets, and oral films [1]. NIR spectra were used in the PLS regression, and the models were cross-validated using venetian blinds, with paracetamol concentrations in the 4–40% (*w*/*w*) range. Drug distribution and the solid state of the API in the dosage forms were tested via Raman confocal microscopy, further confirmed by XRPD analysis. The study revealed a homogeneous drug distribution in which the API was in amorphous phase, present as a solid dispersion, which could promote drug bioavailability and dissolution. The PLS model built for the prediction of paracetamol concentration from NIR spectra showed excellent linearity and accuracy, and paracetamol concentration could be determined regardless of the printlet shape by using the same prediction model [1]. Although transferability across dosage forms is an important step forward, model transferability between different printers remains to be evaluated.

Another limitation of current PAT approaches is the limited evaluation of multi-API dosage forms. Given that 3D printing enables the incorporation of multiple drugs into a single dosage form to enhance patient adherence, reliable simultaneous quantification is essential. Trenfield et al. studied the possibility of assessing drug concentration using PAT tools in SLS-produced printlets and oral films loaded with amlodipine and lisinopril [106]. Printlets and oral films were analysed using a portable NIRS for API quantification. Both calibration models showed excellent linearity (R^2^ > 0.99) and accuracy (RMSEP = 0.24% in both models), proving the possibility to assess API concentration in a multi-component printlet [106]. Although both APIs were successfully quantified, geometry transfer increased prediction errors (RMSEP = 0.77%), particularly for lisinopril, indicating the need for more robust calibration models.

Not only can the API content and dosage form design vary in 3D-printed medicines, but the polymeric matrix can also differ considerably. Trenfield et al. used NIRS and Raman spectroscopy for the quality control of amorphous itraconazole in SLS printlets [105]. Different polymer grades were investigated to assess the influence of particle size on printability and to evaluate the feasibility of the developed calibration models. PLS regression models were constructed using itraconazole concentrations ranging from 0 to 20% (*w*/*w*). In the NIR spectra, peak morphology and intensity varied according to the crystalline itraconazole content, whereas Raman signal intensity increased with increasing amorphous itraconazole content. Both NIR and Raman models demonstrated excellent linearity and predictive performance (RMSEP_NIR_ = 1.04%; RMSEP_Raman_ = 0.63%) [105]. However, the study did not assess model transferability across dosage forms containing different polymer compositions. The different polymer grades were primarily employed to investigate their effects on printability, sintering behaviour, and API solid-state properties. Evaluating model transferability across formulations containing different excipients would represent an important step toward the development of more broadly applicable PAT strategies.

The above-mentioned studies only assessed API content and solid state; none of them concentrated on the assessment of other CQAs. Therefore, Trenfield et al. studied the release profiles of SLS printlets, prepared at different laser scanning speeds, by NIRS. With laser scanning speed increasing, drug dissolution was higher, due to the increased porosity and lower density. Univariate models were created for the density and for the API release predictions, both models exhibiting acceptable linearities (R^2^ > 0.93) [108]. However, the study analysed five tablets as a test set and three time points measured for dissolution, which raises concerns over the real-world implementation.

### 4.4. Light-Based Technologies

#### Stereolithography

Although SLA enables the fabrication of dosage forms with extremely fine details due to its ability to produce very thin cross-sectional layers, the limited availability of biocompatible materials may explain the relatively low number of studies focused on this printing technique. To the author’s knowledge, only a limited number of articles have assessed the use of modern imaging tools for the quality evaluation of SLA-printed dosage forms [14,44]. The lack of studies regarding spectroscopic methods as PAT tools is due to the fact that the surface of SLA printed products is glossy, which majorly distorts spectral analysis.

However, Robles-Martinez et al. used Raman chemical imaging to study the API distribution and solid-state in a printlet containing six APIs produced via SLA 3DP [117]. Through Raman spectra, they could prove the diffusion of one API onto the other layer, and they could also see the layer with the six APIs, which proved that polypill making can be achieved by SLA [117]. Although here, Raman CI has not been used as a PAT tool, it could assess the API distributions within each layer.

Pilch et al. developed a low-cost luminescent sensor-based printlet surface mapping system [116]. 3D printlet surfaces were marked with a luminescent compound with a wide absorbance spectrum. Reflected light from the labelled printlet surface was recorded using a camera, enabling visualisation of the luminescence-marked three-dimensional surface inscriptions. Thus, this study showed the feasibility of a unique, low-cost method that could possibly be applied in the QC assessment of 3D-printed products. However, it remains unclear from the article whether the printlets, following luminescence-based analysis, were intended for patient administration [116].

### 4.5. Overview of Process Analytical Technology in Pharmaceutical 3DP

PAT tools, such as NIRS and Raman spectroscopy, offer rapid evaluation of individual samples without requiring sample preparation. These techniques can be implemented in-line, through handheld devices, at various stages of the fabrication process. They enable real-time monitoring of intermediate CQAs, such as API dispersion in filaments or pastes used for FDM and SSE 3DP. Additionally, PAT tools can be employed for the assessment of final product CQAs, including drug content uniformity and dissolution profile.

However, it should be mentioned that a vast number of the published studies have primarily applied the spectroscopic techniques as QC tools for final product quality, rather than for real-time monitoring and control of the CPPs during the manufacturing process. This highlights an important gap between the demonstrated analytical capabilities of PAT tools and their full implementation within a QbD-driven, closed-loop manufacturing framework.

**Figure 2:**
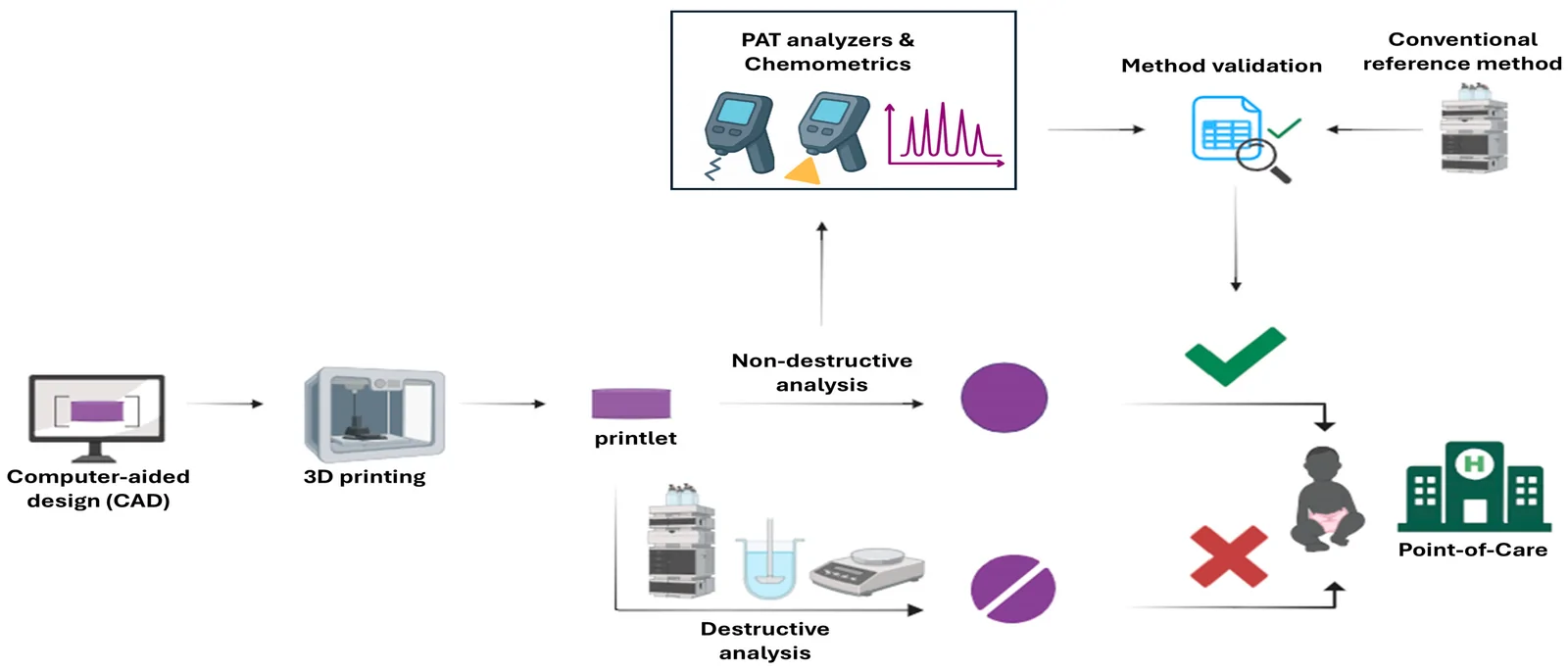
PAT instruments implementation into the PoC.

The principal advantage of PAT tools lies in their ability to support the immediate use of printed dosage forms at the PoC. Thus, they represent a key enabler for the regulatory acceptance and practical implementation of pharmaceutical 3DP in clinical settings [7,146]. Figure 2 gives an overview approach from the design development through to the implementation possibility at the PoC.

## 5. Current Landscape of PAT Applications in Pharmaceutical 3DP

In the previous chapters, each printing technology had been discussed separately, identifying the main CPPs influencing the CQAs of printlets and exploring how these CQAs could be monitored using non-destructive PAT methods. Findings indicate that the application of PAT tools has been predominantly applied in the QC of FDM, IJP and SLS 3D-printed dosage forms, as reflected by the number of published studies. Conversely, a notable lack of PAT-related research can be observed predominantly for BJ and SLA, as summarised in Table 2, despite their demonstrated feasibility to produce personalised medicines.

According to the available literature, printlet QC is commonly performed using conventional analytical techniques, such as UV–Vis spectroscopy and HPLC for drug content determination and thermal analysis techniques for solid-state determination of API. These techniques are also used as reference methods for the validation of the developed QC analysis by PAT tools.

The currently existing examples of PAT implementation in various printing technologies have been summarised in Table 5. As shown, the predominant PAT tools employed are NIR reflection spectroscopy and Raman spectroscopy, often coupled with imaging techniques [48,49,143]. Although these spectroscopic tools can provide comprehensive information on multiple quality parameters, most studies have primarily focused on assessing drug content and spatial drug distribution, with a relatively limited number of studies addressing CQAs, such as the in vitro dissolution profiles of the printlets.

Therefore, the synthesis presented in Table 5 highlights both the successful integration of PAT tools into the QC of personalised medicine and the need for their broader application in multiple CQAs monitoring.

Most studies have focused on the development of individual calibration models, whereas the current state of PAT-based API quantification in pharmaceutical 3D printing is limited. An overview of the available studies is presented in Table 6. Each individually developed API quantification model, validated within a single formulation and a single printer, consistently achieved low prediction errors and high linearity across all printing technologies reviewed. Where directly comparable, NIR and Raman spectroscopy produced similar predictive accuracy—Trenfield et al. [105] reported RMSEP values of 1.04% and 0.63% for NIR and Raman, respectively, in itraconazole solid-state form prediction—though the two techniques exploit different spectral information and this equivalence should not be generalised beyond the specific formulations studied.

Cross-study comparison is fundamentally limited by inconsistent validation reporting. Several studies report only RMSEC or RMSECV, which are in-sample metrics that systematically overestimate predictive accuracy; RMSEP is the only appropriate measure of true predictive performance and is absent from a substantial proportion of the reviewed literature. There is additionally no unified unit convention, with some studies reporting RMSEP in absolute mass (µg, mg) and others in %*w*/*w*, preventing any meaningful numerical comparison across entries. The broader regulatory context highlights this problem. ICH Q2(R1), the EMA and the FDA NIR guidance [161,162,163] collectively provide a validation framework covering specificity, linearity, accuracy, and precision, but all three documents were designed for conventional batch manufacturing and none specifies acceptable RMSEP ranges, maximum permissible latent variable (LV) counts, required preprocessing approaches, or minimum validation sample sizes for models applied to single-unit fabrication with inherently variable physical properties. The practical consequence is that acceptability thresholds are set by individual research groups without external reference—one group’s 0.5% RMSEP defines the benchmark while another accepts 1.5% with equal confidence. Establishing technology-specific validation thresholds for multivariate API quantification models in pharmaceutical 3D printing is an unaddressed regulatory priority.

No PAT model in the reviewed literature has been validated across different printers of the same type or transferred across printing technologies. The reasons are physical rather than logistical. NIR and Raman spectra simultaneously encode chemical and physical information, and between printers of the same type, differences in laser power calibration (SLS), nozzle temperature profiling (FDM), or extrusion pressure consistency (DPE/SSE) produce tablets with differing surface morphologies and densities—differences that generate spectral variance indistinguishable from drug concentration changes within a model not explicitly trained on multi-printer samples. Trenfield et al. [108] demonstrated this physical sensitivity directly: varying SLS laser scanning speed between 100 and 180 mm/s produced a systematic and linear shift in NIR baseline absorbance at 9000 cm^−1^, driven entirely by changes in tablet density and porosity rather than drug content. An API quantification model calibrated at a fixed laser speed would be exposed to this spectral variance if applied at a different speed, influencing drug content prediction independently of any true concentration change. The same principle applies across printing technologies, where each process produces a fundamentally different tablet microstructure that a calibration set designed for one technology cannot represent.

Moreover, all reviewed models were built on single drug–polymer–excipient combinations. PLS and OPLS regression models capture variance patterns specific to the spectral matrix of the calibration formulation and are not transferable to a different API even when the printing process, geometry, and concentration range are held constant—a model built on a specific API encodes the API-specific spectral features and has no predictive basis for other API. Broadening calibration sets to include multiple drugs and excipients would improve same-technology transferability but would not resolve the physical presentation problem described above. Tablet geometry is a further underexplored aspect. NIRS absorbance is sensitive to surface curvature and roughness. Since geometry customisation is one of the primary clinical arguments for pharmaceutical 3D printing, the limited geometry transferability assessments across the reviewed literature represent a direct contradiction of the technology’s stated purpose.

**Table 5:** Current PAT analysis applied in pharmaceutical 3D printing QC.

Printing Method	API	Analytical Method	Property Assessed	D Osage Form	Model Used	Reference Method	References
**FDM**	Metoprolol tartrate	Raman spectroscopy	Drug content Solid state analysis	Filaments	PLS	DSC analysis	[80]
	Metoprolol tartrate	Raman spectroscopy	Drug content	Solid oral printlet	PLS/MCR	-	[153]
	Tamoxifen	NIR diffuse-reflectance	Drug content	VCM tablet surrogates, solid oral printlets	PLS	HPLC	[5]
	Hydrocortisone	NIR reflectance	Drug content	Solid oral printlet	PLS	HPLC	[152]
	Caffeine	NIR reflectance	Drug content	Solid oral printlet	PLS	HPLC	[86]
	Nitrofurantoin/Indomethacin	NIR-HIS	Drug content Excipient content Spatial distribution of API	Oral thin films	MCR-ALS	-	[157]
	Saquinavir, halofantrine	µCT, TPI	Porosity	Solid oral printlet	-	-	[85]
**SSE**	Furosemide	NIRS reflectance	Blend uniformity	Solid oral printlet	PLS	HPLC	[94]
**DPE**	Efavirenz	NIRS reflectance	Drug content	Solid oral printlet	PLS	HPLC	[38]
**IJP**	Prednisolone/Levothyroxine	NIRS reflectance	Drug content	Oral thin films	PCA, OPLS	HPLC	[114]
	Metformin hydrochloride	NIRS-HIS	Drug content Spatial distribution of API	Solid oral printlet	PCA, PLS	HPLC	[49]
	Anhydrous theophylline	NIRS-HIS	Drug content Spatial distribution of API	Solid oral printlet	PCA, PLS	UV spectroscopy	[48]
	Caffeine/Loperamide	ATR-FTIR	Drug content	Solid oral printlet	PLS	UV–Vis spectroscopy→caffeine HPLC→ loperamide	[142]
	Propranolol/Montelukast/Haloperidol	NIR-transmission Raman spectroscopy	Drug content	Solid oral printlet	PLS	HPLC	[113]
	Haloperidol/Acetaminophen	Raman CI	Drug content Spatial distribution of API	Solid oral printlet	PCA, PLS	HPLC	[146]
	Vitamin B1, B2, B3 and B6	Colorimetry	Drug content	-	-	LC-MS	[111]
**SLS**	Acetaminophen	NIR reflectance Raman CI	Drug content, Spatial distribution of API	Solid oral printlet	PLS	HPLC XRPD	[1]
	Amlodipine + Lisinopril	NIR-reflectance	Drug content	Polyprintlets	PLS	UV–Vis spectroscopy, HPLC	[106]
	Itraconazole	NIR-reflectance Raman spectroscopy	Amorphous drug content	Solid oral printlet	PLS	HPLC	[105]

**Table 6:** Currently existing PAT studies on API quantification in 3DP.

Printing Method	API	Concentration Range	Excipient	PAT Tool (in/on/at-Line)	Calibration/Validation Errors	R^2^(Pred)	Model Type & LVs	Preprocessing	Validation Approach	Transferability Tested	References
FDM	Caffeine	0–40% *w*/*w*	PVA, sorbitol	NIRS (in-line)	RMSEP: Full: 1.40%; 20%: 2.22%; 40%: 1.65%; 80%: 1.41%	Full: 0.985 Others: 0.983–0.991	PLS; various LVs	2nd derivative (SG)	Specificity; accuracy; paired t-test; precision	-	[86]
	Hydrocortisone	0–15% *w*/*w*	Not stated	NIRS (in-line)	RMSECV = 0.46%	0.981	PLS; LVs not stated	SNV, SG	Specificity, linearity; accuracy precision (RSD%)	-	[152]
	Nitrofurantoin anhydrate	Not stated	Polylactic acid, hydroxyapatite	NIRS-HIS (off-line)	Not stated	Not stated	MCR-ALS (semi-quantitative; no formal PLS calibration)	SNV, SG	No formal validation	-	[157]
SSE	Furosemide	1–2% *w*/*w*	Polysorbate, excipient base	NIRS (at-line)	Not reported RMSEC = 3.37%, RMSECV = 3.68%	0.91	PLS; LVs not stated	1st derivative, SNV	Accuracy; linearity; precision (RSD%); robustness	-	[94]
DPE	Efavirenz	0–35% *w*/*w*	Kollidon VA 64, Mannitol 100	NIRS (in-line)	Not reported RMSE = 1.07%	0.983	PLS; LVs not stated	2nd derivative, SG	Not stated	-	[38]
	Theophylline	Fixed dose of approximately 19.5% (*w*/*w*)	Klucel JF, D-mannitol	Transmission Raman spectroscopy (not stated)	RMSEC = 0.422 RMSECV = 0.624 RMSEP= 0.5611%	0.977	PLS; LVs not stated	Whittaker, SNV	Paired t-test	-	[88]
IJP	Levothyroxine Prednisolone	Prednisolone: 0.433–1.951 mg Levothyroxine: 0.148–0.859 mg	HPC, HPMC, glycerol; substrate: edible icing sheet or HPC + HPMC + glycerol film	NIRS (in-line)	RMSEP not reported RMSECV: prednisolone 0.056–0.069 mg levothyroxine 0.051–0.061 mg	0.948–0.990	OPLS, LVs not stated	MC, SG, 3rd derivative	Recovery (%)	Substrate type: 2 types tested	[160]
	Metformin HCl	10–500 drops/spot (calibration); multilayer test samples 1–30 layers; HPLC reference range 1–90 µg/mL; API mass per spot up to ~800 µg	Gelatin film strips with 2% titanium dioxide	NIR-HIS (Raman CI reference) (not stated)	Not stated	Not stated	PLS-5LVs	SG, SNV	Pre-validation only	Not tested	[164]
	Theophylline	7.5 µg cm^−2^ per printing pass	Glycerol, water, ethanol; substrate: copying paper	NIRS -HIS (not stated)	Not stated	0.9994	Not stated	SNV	Not stated	-	[48]
	Anhydrous caffeine Loperamide HCl	Not stated	PG + water; substrate: Polyethylene terephtalate (PET) film	ATR-FTIR (at-line)	RMSEP: 0.19–0.76 (units not stated)	0.99	Not stated	SNV	Not stated	Printing resolution: different resolutions tested	[142]
	Propranolol Haloperidol Montelukast	0.5–4.1 mg 0.6–4.1 mg 2.1–12.1 mg	HPMC porous substrate	NIRS + Raman spectroscopy	RMSEP: 0.15–0.86 mg	0.95–0.99	PLS; LVs not stated	Not stated	Not stated	-	[113]
	Haloperidol	0.09–2.73 mg	CaHPO_4_ compacts/ paracetamol tablets/ HPMC foam	Raman spectroscopy (not stated)	RMSEP: CaHPO_4_: 52 µg	0.97–0.99	PLS; LVs not stated	SG, SNV	Not stated	Substrate: 3 types tested	[146]
SLS	Paracetamol	4–40% *w*/*w*	Eudragit L100-55 (external test: HPMC E5)	NIRS (not stated)	RMSEP: 0.63%	0.996	PLS, 3 LVs LV1 = 88.8%	2nd derivative SG, MC	Specificity; linearity; accuracy paired t-test; precision (RSD); intermediate precision (RSD)	Geometry Excipient: Partial	[1]
	Amlodipine Lisinopril	1–5% *w*/*w* 2–10% *w*/*w*	PEO 100,000 Candurin Gold Sheen	NIRS (not stated)	RMSEP_lisinopril_ = 0.24%; RMSEP _amlodipine_0.24%;	0.997; 0.991	PLS LVs not stated	Amlodipine: 2nd derivative SG, MSC, MC Lisinopril: 2nd derivative SG, SNV + MC	Specificity; linearity; accuracy; paired t-test	Geometry (films→cylindrical)	[106]
	Itraconazole (20% *w*/*w* fixed drug load)	0–20% *w*/*w*	HPC-L, HPC-SL, HPC-SSL	NIRS Raman spectroscopy	RMSEP NIRS: 1.04% RMSEP Raman: 0.63%	NIRS: 0.998 Raman: 0.993	PLS, LVs not stated	NIRS: 2nd derivative + MC Raman: Whittaker + MC	Specificity;Linearity; Accuracy	-	[105]

LV = latent variable; MC = mean centering; MCR-ALS = Multivariate Curve Resolution–Alternating Least Squares; OPLS = Orthogonal Partial Least Squares; PLS = Partial Least Squares; RMSE = Root Mean Square Error; RMSECV = Root Mean Square Error of Cross-Validation; RMSEP = Root Mean Square Error of Prediction; RSD = Relative Standard Deviation; SG = Savitzky–Golay; SNV = Standard Normal Variate.

No reviewed study has assessed whether a calibration model remains valid beyond the immediate post-printing period. ICH Q2(R1) identifies conditions requiring revalidation—including changes in analytical procedure, process, or product specification, and the EMA NIRS guideline [161,163] acknowledges the need for ongoing model maintenance, but neither document provides a framework applicable to 3D printing environments, where model drift may arise from laser power degradation over time, inter-batch variability in polymer particle size distribution, and environmental changes in ambient humidity and temperature affecting both the spectrometer and the powder feedstock. In the absence of any published long-term stability data, the durability of NIRS and Raman calibration models under real operational conditions over weeks or years of use remains entirely uncharacterised.

## 6. Regulatory Hurdles of 3DP and Future Perspectives

### 6.1. United States Food and Drug Administration

Following the 2015 approval of Spritam^®^ (levetiracetam, Aprecia Pharmaceuticals) as the first and only market-authorised 3D-printed pharmaceutical product globally, regulatory interest in additive manufacturing technologies grew considerably, yet no clear product-specific guidance has followed [165]. Products manufactured through 3DP continue to be assessed under 21 CFR Parts 210 and 211, batch-oriented cGMP regulations containing no supply for printer qualification, pharma ink stability, or the sensitivity of spectroscopic calibration models to printing process parameter variation [166]. The basis for PAT in US pharmaceutical manufacturing was established by the 2004 guidance PAT “A Framework for Innovative Pharmaceutical Development, Manufacturing, and Quality Assurance”, which positioned timely CQA measurement as the mechanism through which process understanding replaces end-product testing and introduced real-time release as a data-driven quality assurance strategy [10]. This guidance remains the primary US regulatory reference for spectroscopic in-process monitoring and has not been updated since publication. NIR-based analytical procedures fall under the 2015 guidance Development and Submission of Near-Infrared Analytical Procedures, which references ICH Q2(R1) and USP <856> but was written without consideration of single-unit fabrication, PoC implementation, or the physical property sensitivity effects documented throughout 3DP PAT literature [10]. The Framework for Regulatory Advanced Manufacturing Evaluation (FRAME) initiative produced a discussion paper in October 2022 “Distributed Manufacturing and Point-of-Care Manufacturing of Drugs” identifying regulatory challenges of PoC manufacturing inclusive of 3DP, without regulatory guidance [167] A January 2025 draft guidance on 21 CFR 211.110 acknowledges advanced manufacturing technologies including 3DP in the context of in-process testing, again without technology-specific QbD or PAT provisions [168]. Design space submission, Real-Time Release Testing approval, and PAT calibration model lifecycle management for 3D-printed drug products consequently remain without a defined regulatory pathway.

### 6.2. European Medicines Agency

The EMA’s “Questions and Answers on the Implementation of 3DP Technology (Additive Manufacturing Technology) for Solid Oral Dosage Forms” is the most technically detailed regulatory position on quality and GMP requirements for pharmaceutical 3DP published by any major regulatory body [169]. The document supports ICH Q8(R2) as the applicable development framework, recommends formal design space establishment for both pharma ink characterisation and printing process development, and identifies printing-specific CPPs, including: nozzle geometry, head and chamber temperatures, extrusion pressure, printing speed, and jetting frequency; as requiring systematic characterisation against CQAs including content uniformity, dissolution, porosity, solid-state form, assay, degradation products, and stability [170]. NIR and Raman spectroscopy are recognised as suitable PAT tools for in-process control, and the EMA Guideline on Real-Time Release Testing is referenced as the regulatory basis for replacing end-product testing with process-data-driven release decisions [171]. Process validation is classified as non-standard under Annex II of the EMA process validation guideline; when technology is transferred from printer or pharma ink manufacturer to an end user site, the end user must demonstrate process robustness and reproducibility through dedicated confirmatory batches [169]. The document, however, remains silent on two aspects of direct relevance to PAT implementation. It does not clarify whether chemometric calibration models supporting NIRS or Raman-based control must be independently revalidated at each receiving site, or whether a centrally developed model may be transferred without additional qualification. It equally provides no quantitative acceptance criteria, no RMSEP thresholds (as discussed in the previous chapter), precision specifications, or minimum latent variable requirements against which model suitability can be assessed. In the absence of such specifications, acceptance decisions fall to individual investigators and manufacturers, producing heterogeneous reporting and limited cross-study comparability. As this document postdates the majority of PAT-focused 3DP publications, future studies addressing calibration transfer and multi-site model performance would be substantially strengthened by clearer regulatory expectations and would support broader clinical adoption of PAT-enabled 3DP.

### 6.3. UK Medicines and Healthcare Products Regulatory Agency

The MHRA has produced the most legally advanced regulatory instrument for pharmaceutical 3DP of the three bodies reviewed. The Human Medicines (Amendment) (Modular Manufacture and Point-of-Care) Regulations 2025, entering UK law on 23 January 2025 and in force from 23 July 2025, introduce PoC medicines, within which 3D-printed products that can only be manufactured at the PoC are explicitly defined, and modular manufacturing medicines as new regulatory categories under the Human Medicines Regulations [172]. This development supports a more patient-centred approach to treatment and supports the feasibility of integrating 3DP technologies into clinical settings at the regulatory level [173]. Its adoption runs parallel to advances in GMP-compliant pharmaceutical hardware: the M3DIMAKER 1 and M3DIMAKER 2 systems developed by FABRX incorporate multiple exchangeable extrusion nozzles and an integrated balance for in-line mass measurement as unit-level QC, designed for deployment in hospital and community pharmacy settings [174]. Quality and GMP requirements for PoC-manufactured products operate through EU GMP—adopted as UK GMP following Brexit—incorporating ICH Q8(R2) and Q2(R1) through existing cross-references without 3DP-specific extensions [163,170]. No published MHRA guidance addresses design space construction, PAT tool validation criteria, Real-Time Release Testing submission requirements, or PAT calibration model lifecycle management for 3D-printed products in a PoC setting, leaving the framework legally advanced in establishing the product category and licencing pathway while technically silent on the implementation standards those operating within it must meet.

### 6.4. QbD Framework, PAT Strategies, and Point-of-Care Implementation

The three regulatory frameworks reviewed above collectively establish that, while the legal and procedural infrastructure for PoC pharmaceutical 3DP is developing, the technical requirements governing QbD-aligned process design and validated PAT implementation remain partially or wholly unspecified. Meeting these requirements in practice demands both hardware capable of operating under pharmaceutical quality standards and analytical strategies capable of verifying product quality non-destructively at the point of manufacture.

**Figure 3:**
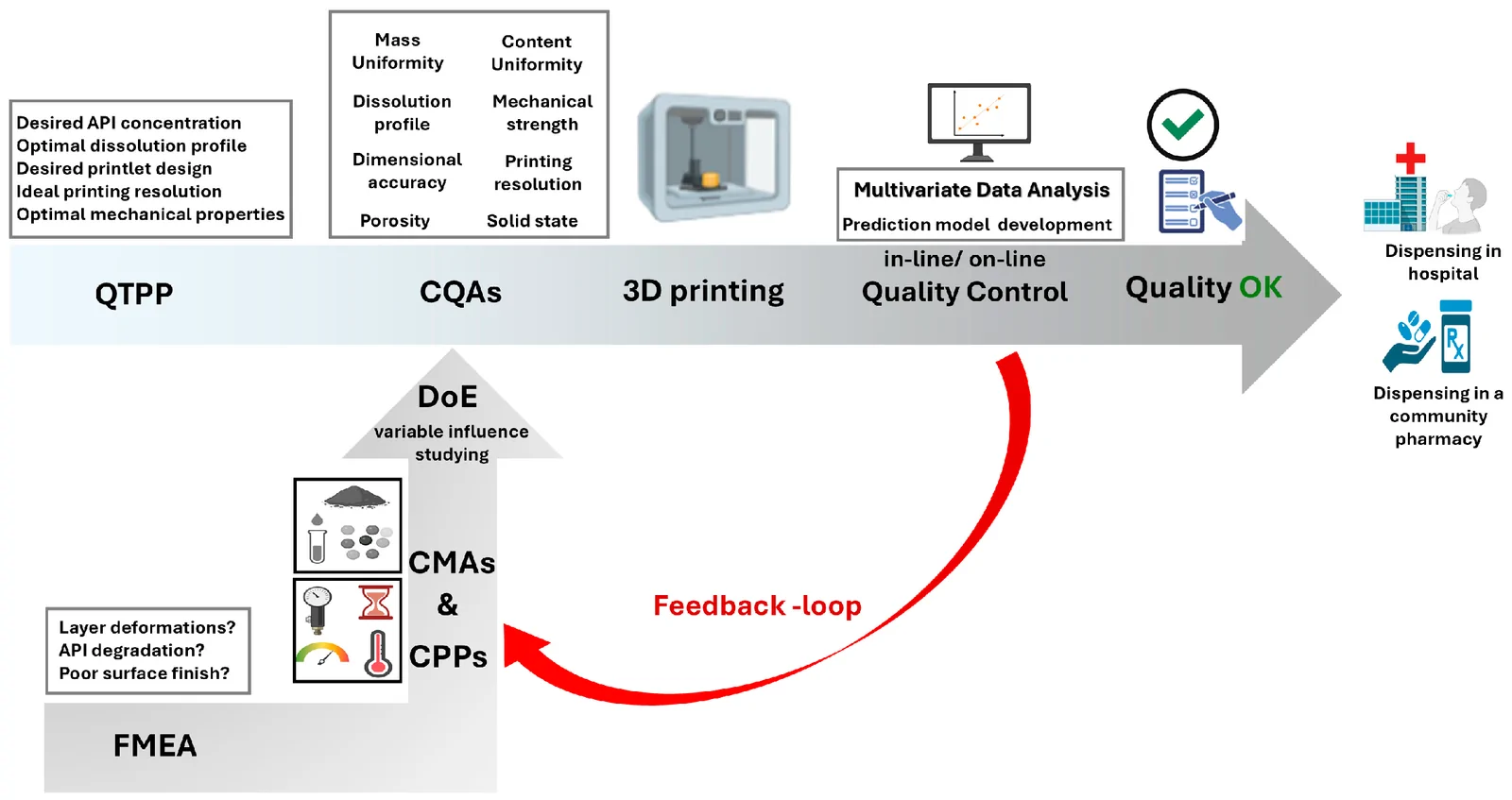
QbD framework and PAT implementation in PoC manufacturing of 3D-printed medicine.

Conventional pharmaceutical QC relies predominantly on end-product testing and trial-and-error approaches that offer limited insight into process variability and no basis for real-time intervention [175]. Such strategies are structurally incompatible with PoC 3DP, where rapid decision-making and thorough process understanding are prerequisites for safe operation rather than optional features. Historically, insufficient knowledge of CPPs and material attributes has driven batch failure and non-compliance in pharmaceutical manufacturing, and the same risk applies to 3DP if quality assurance is deferred to post-production testing. QbD addresses this directly by embedding quality into product and process design rather than relying on retrospective verification [176]. Implementation begins with systematic risk assessment, using Failure Mode Effect Analysis (FMEA) tools (e.g Ishikawa diagrams) to identify factors capable of compromising product quality, followed by clear definition of the QTPP and corresponding CQAs. Design of Experiment (DoE) studies are subsequently conducted to identify and quantify the CPPs most influential on printed product quality for the specific technique, formulation, and target patient population under study [175,176,177]. This structured characterisation produces the process understanding on which a formal design space can be established and within which a PAT-based control strategy can operate, as illustrated in Figure 3.

The development of GMP-compliant 3D printers adequate for PoC deployment remains a primary obstacle in this process. Most commercially available platforms were engineered for industrial or prototyping applications, and their adaptation to pharmaceutical quality standards, covering cleanability, cross-contamination control, software validation, and audit-trail generation, demands both hardware redesign and compliance work [17]. Examples confirm that such adaptation is achievable. The M3DIMAKER 2 pharmaceutical printer developed by FABRX resolves cross-contamination through the incorporation of blister packaging within the printing cycle and the use of disposable syringes, producing a GMP-compliant SSE system [174]. In a different technology context, Robles-Martinez et al. adapted a commercial SLA printer to fabricate a six-layer polypill containing six APIs, requiring modification of printer software to allow automatic elevation of the build platform during pauses in the print cycle, thereby enabling sequential resin changes that the original design prevented [117]. The PolyPrint consortium is pursuing equivalent work for FDM 3DP, with the aim of producing platforms suitable for pilot clinical studies at the PoC [17].

PAT tools enable real-time, non-destructive CQA monitoring during manufacture and, when integrated with feedback control systems, allow automatic CPP adjustment to maintain the process within the validated design space. Meeting the requirements of ICH Q8, Q9, and Q10 in a PoC 3DP setting demands analytical tools that are robust across the variability inherent in Point-of-Care environments [86,178]. Developing such tools remains technically demanding. Detection of low-dose APIs, accurate quantification in multi-drug formulations, and the peak separation of spectral contributions from excipients or carrier substrates (particularly problematic in inkjet-based systems) each represent unresolved challenges that limit the generalisability of currently published models. Advanced chemometric algorithms incorporating non-linear regression and 3DP-specific preprocessing strategies offer partial solutions, and when combined with feedback-enabled control architectures they hold potential for real-time CPP adjustment maintaining CQAs within predefined limits with minimal human intervention, a capability directly relevant to consistent dosage personalisation in clinical settings [175].

Three principal implementation architectures have been proposed for pharmaceutical 3DP in hospital and community pharmacy settings. The first involves complete on-site manufacturing, where raw materials are supplied, and both printing and QC are conducted locally. The second relies on pharma ink or feedstock preparation by a pharmaceutical company, with printlet fabrication and QC performed at the PoC—the model most directly supported by the MHRA framework and the EMA’s technology transfer provisions [169,173]. The third is centralised manufacturing, in which both feedstock and finished printlets are produced by the pharmaceutical company and distributed to dispensing sites [7]. Each model carries distinct regulatory implications for the transfer of validated PAT calibration models between manufacturing and dispensing locations, and regardless of the approach, a validated PAT strategy appropriate to the printing technology and formulation is a prerequisite for quality assurance throughout.

Future perspectives: The advancement of pharmaceutical 3DP toward routine PoC use will depend on progress across four interrelated fronts: computational design, digital infrastructure, materials and quality control, and economic sustainability.

Computationally, QbD frameworks are increasingly incorporating generative artificial intelligence, including generative adversarial networks and reinforcement learning, to predict optimal drug–excipient combinations and reduce the experimental burden of formulation development for APIs not yet characterised in 3DP [179,180]. The same trajectory extends to manufacturing, where deep neural network control systems optimise process parameters in real time from historical data to improve process stability and resource utilisation [181]. Limitations in model robustness, interpretability, and computational demand currently restrict routine use [180,181], but machine learning is expected to be progressively integrated into 3DP workflows, enabling precise design of dosage forms with defined CQA profiles [182]. Realising this capability at the PoC in turn requires secure digital infrastructure: cloud-based storage of digital models, process parameters, and production records, with protection of patient-specific data, while unit-level QR codes linked to production and quality records provide traceability against dosing or design mix-up [180].

These computational advances nonetheless remain bounded by material and analytical constraints. Excipients are limited and selected primarily for printing properties; thermally sensitive drugs are problematic in melt-based techniques such as FDM, and many photopolymer and laser-sintering materials fall outside the FDA Generally Recognised As Safe (GRAS) list, precluding use in marketed dosage forms [183]. Batch-to-batch uniformity is further compromised by variability in materials, printer settings, and ambient conditions, with morphological inconsistencies capable of altering solubility and bioavailability [183]. Consequently, wider hospital adoption is expected to depend on commercial, at least partially GMP-compliant, pharmaceutical inks; without them, every formulation must be individually validated [62]. For characterised inks, a validated GMP-compliant printer with gravimetric control may suffice, whereas for less-characterised inks NIR can serve as a PAT tool for identification and content determination, given a validated model per API and formulation [62].

Underlying all of these is the question of economic sustainability. Evidence from conventional manufacturing indicates that PAT restructures rather than adds to quality control costs, shifting assurance from finished-product testing to continuous in-process monitoring and reducing waste, manufacturing time, and rejects [133]. The resulting benefits extend across stakeholders: patients gain individualised doses and modified-release profiles at higher quality [184]; healthcare systems may reduce macro-level costs through improved therapeutic control and per-unit manufacturing costs fall with utilisation and economies of scale [184]. Together with the absence of standardised regulatory frameworks [183] and the scarcity of formal costing studies [184], these factors define robust pharmacoeconomic evaluation and clearer regulatory pathways as the central prerequisites for both industrial and regulatory adoption.

## 7. Conclusions

As interest in Point-of-Care pharmaceutical 3D printing continues to grow, the demand for robust, non-destructive, and analytically rigorous QC strategies becomes increasingly critical to its broader clinical implementation. While regulatory bodies, including the FDA, EMA, and MHRA, are actively developing dedicated frameworks (most notably the EMA’s 2026 Q&A and the MHRA’s 2025 Point-of-Care legislation), the absence of standardised, technology-specific QC requirements remains one of the principal barriers to routine clinical adoption. A key advancement in addressing this gap lies in the systematic integration of a QbD framework into the 3DP manufacturing process, whereby the QTPP, CQAs, and corresponding CPPs are clearly defined, monitored, and controlled for each printing technology and formulation type, since without this structured process, understanding the consistent production of high-quality personalised dosage forms cannot be reliably achieved. Although recent research has increasingly focused on NIR and Raman spectroscopy as PAT tools for non-destructive CQA assessment in 3D-printed dosage forms, the number of studies covering the full range of clinically relevant CQAs remains limited, and no published model has demonstrated transferability across different printers or manufacturing sites. Further work is therefore needed to optimise chemometric data processing approaches and establish validated, reproducible QC protocols that meet regulatory expectations, including the definition of acceptable prediction error thresholds and model revalidation criteria currently absent from all three regulatory frameworks reviewed. Closing these analytical and regulatory gaps in parallel, through multi-site PAT model validation, GMP-compliant printer development with integrated feedback control, and the establishment of technology-specific guidance extending existing Real-Time Release Testing and NIR/Raman frameworks to the 3DP context, represents the critical path for pharmaceutical 3D printing to transition from a well-evidenced research platform to a clinically deployable standard of personalised care.

## Figures and Tables

**Table 4:** Overview of NIRS, Raman spectroscopy and ATR-FTIR [129,136,142].

Characteristics	NIRS	Raman Spectroscopy	ATR-FTIR
**Penetration depth**	Low (reflection)/High (transmission)	Low	Low
**Sample volume (mm^3^)**	75	10	10 × 10^−9^
**Water sensitivity**	Yes	No	Yes
**Analysis time**	Rapid	Often time-consuming	Rapid
**In-line implementation**	Yes	Yes	No
**Spectral preprocessing**	Needed	Needed	Needed

*ATR-FTIR = Attenuated Total Reflection–Fourier Transform Infrared spectroscopy.

## Data Availability

No new data were created or analysed in this study. Data sharing is not applicable to this article.

## Abbreviations

The following abbreviations are used in this manuscript:

3DP	Three-Dimensional Printing
AET	Acoustic Emission Technology
API	Active Pharmaceutical Ingredient
AsLs	Asymmetric Least Squares Smoothing
ATR-FTIR	Attenuated Total Reflectance Infrared Spectroscopy
BJ	Binder Jetting
CAD	Computer-aided Design
CI	Chemical Imaging
CMAs	Critical Material Attributes
CPPs	Critical Process Parameters
CQAs	Critical Quality Attributes
DoE	Design of Experiments
DoD	Drop on Demand
DPE	Direct Powder Extrusion
DSC	Differential Scanning Calorimetry
EDX	Energy-Dispersive X-Ray Microanalysis
EMA	European Medicines Agency
FDA	Food and Drug Administration
FDM	Fused Deposition Modelling
FMEA	Failure Mode and Effects Analysis
GMP	Good Manufacturing Practice
GRAS	Generally Recognised as Safe
HME	Hot Melt Extrusion
IJP	Inkjet Printing
IR	Infrared
LV	Latent Variable
LC-MS	Liquid Chromatography–Mass Spectrometry
MHRA	Medicines and Healthcare products Regulatory Agency
MSC	Multiplicative Signal Correction
MCR-ALS	Multivariate Curve Resolution–Alternating Least Squares
MVDA	Multivariate Data Analysis
NIRS	Near-Infrared Spectroscopy
NIRS HIS	Near-Infrared Spectroscopy Hyperspectral Imaging
NMR	Nuclear Magnetic Resonance
OPLS	Orthogonal Partial Least Squares
PAT	Process Analytical Technology
PIJ	Piezoelectric Inkjet
PLS	Partial Least Squares
PoC	Point of Care
RMSECV	Root Mean Square Error of Cross-Validation
RMSEP	Root Mean Square Error of Prediction
QbD	Quality by Design
QC	Quality Control
QTPP	Quality Target Product Profile
SEM	Scanning Electron Microscopy
SLA	Stereolithography
SLS	Selective Laser Sintering
SNV	Standard Normal Variate
SSE	Semisolid Extrusion
SWLI	Scanning White Light Interferometry
TGA	Thermogravimetric Analysis
TIJ	Thermal Inkjet
Tg	Glass Transition temperature
TPI	Terahertz Pulsed Imaging
UV	Ultraviolet
VCM	Vacuum Compression Moulding
XRPD	X-Ray Powder Diffraction
µ-CT	X-Ray Microtomography
